# Integrative analysis of metabolome and transcriptome reveals new insights into the formation and regulation of major components of *G. leucocontextum* and *G. lucidum*

**DOI:** 10.1128/spectrum.01686-25

**Published:** 2026-05-18

**Authors:** Huijuan Sun, Xuefeng Zhu, Junli Zhang, Lei Gao, Gason Robeb, Yuanzuo Lv

**Affiliations:** 1 Institute of Vegetables, Xizang Academy of Agricultural and Animal Husbandry Sciences 232815, Lhasa, Tibet, China; 2 Innovation Center for Edible Fungi Resources and Application Technology,Xizang Autonomous Region, Lasha, China; 3 Bomi County Garonggou Village Forest Resources Cultivation Farmers and Herders Specialized Cooperative Society, Lhasa, Tibet, China; 4 College of Horticulture Science and Technology, Hebei Normal University of Science and Technology 165079, Qinhuangdao, Hebei, China; Punjab University, Lahore, Pakistan; Southwest University, Chongqing, China

**Keywords:** *G. leucocontextum*, *G. lucidum*, RNA-seq, triterpenoids, development

## Abstract

*Ganoderma* is a class of medicinal fungi with diverse medicinal effects, of which triterpenoids are one of the main pharmacologically active substances. In this study, sporocarps of two *Ganoderma* species (*Ganoderma leucocontextum*, a newly discovered species, and *Ganoderma lucidum*, a traditional medicinal species) at five different developmental stages were used as test materials for joint transcriptomic and metabolomic analyses. Metabolomic analysis via ultra-high performance liquid chromatography tandem mass spectrometry identified a total of 514 differential metabolites (screened by VIP ≥ 1 and *P* < 0.05). Among these, triterpenoids were the primary class of differentially abundant metabolites between the two species: *G. leucocontextum* consistently contained higher levels of ganoleucoin during development, while *G. lucidum* maintained higher levels of ganoderic acid. Transcriptomic analysis yielded 227.61 Gb of clean data (*Q*30 base percentage > 93%), with 228,593 significantly differentially expressed genes identified across the five developmental stages. Most genes in the triterpenoid synthesis pathway (including several *Ganoderma*-specific CYP450 family genes) were more highly expressed in *G. lucidum*. RT-qPCR validation of six key DEGs confirmed that their expression trends were consistent with transcriptomic data. Additionally, six putative transcription factors (belonging to HSF and GNAT families) potentially involved in *Ganoderma* triterpene metabolism were identified. Our findings provide data sets on dynamic gene expression and differential metabolites during the development of the two *Ganoderma* species, laying a foundation for subsequent in-depth research.

**IMPORTANCE:**

Since forward genetic dissection of favorable traits is a time-consuming and arduous task in basidiomycete mushrooms, comprehensive analysis using omics technologies has become a feasible option. In this study, five different sporocarp developmental stages (S1–S5) of *Ganoderma leucocontextum* and *Ganoderma lucidum* were selected, and through combined transcriptome and metabolome analysis, we aimed to preliminarily clarify the differences in active ingredients and key genes related to active substances in *G. leucocontextum* compared with traditional *Ganoderma* (*G. lucidum*; Chizhi).

## INTRODUCTION

*Ganoderma* belongs to the kingdom of Fungi, division of Basidiomycota, class of Homobasidiomycetes, order of Aphyllophorales, and family of Polyporaceae (*Ganodermataceae*) (1–3). Although *Ganoderma* fruiting bodies are not classified as edible mushrooms due to their thick and corky texture, they have been widely studied as medicinal mushrooms owing to their rich content of bioactive components, including triterpenes, polysaccharides, nucleosides, and alkaloids (4–6). In traditional Chinese medicine, *Ganoderma* is described as having the ability to tonify the “qi” and calm the spirit, and to relieve coughs and asthma (7). *Ganoderma lucidum* has been shown to have a variety of effects, including antitumor, antihypertensive, antiviral, and immunomodulatory (8, 9). The observed therapeutic effects are primarily attributed to diverse bioactive constituents, notably triterpenoid compounds (10). These secondary metabolites demonstrate multifunctional pharmacological properties, encompassing antitumor activity, hepatoprotective and detoxifying effects, anti-HIV activity, and cholesterol-lowering effects (9, 11, 12). Not only that, but in recent years, more and more studies have shown that *Ganoderma* has medical potential in the fight against cancer as well (13). The intense bitterness in *Ganoderma* comes from the triterpene, and it depends on the strain, cultivation conditions, and manufacturing processes (14).

According to the international fungal name database Species Fungorum (Index Fungorum Home Page, accessed 19 December 2023), there are 421 accepted species in the genus *Ganoderma*. There are many species of *Ganoderma*, but not all of them have medicinal value. In fact, the Pharmacopoeia of the People’s Republic of China (Part 1) (2000, 2005, 2010, and 2015 editions) listed the fruiting body of *Ganoderma lucidum* Karst (Chi Zhi) and *Ganoderma sinensis* as the legal traditional Chinese medicine. *Ganoderma leucocontextum* is a new species discovered in Linzhi, Tibet, China, in 2015 (15). There have also been reports of this species in Pakistan (16). In recent years, new compounds with pharmacological activities such as triterpenes have been isolated from *G. leucocontextum* (17–19). It has been suggested that *Ganoderma leucocontextum* contains special triterpenoids and polysaccharide components not found in traditional varieties of *G. lucidum*, and that it has the ability to regulate immune activity and other effects (20–22). Although the pharmacological activities have been demonstrated, the genes and metabolic pathways involved in the crucial stages of *G. leucocontextum* are still incomplete due to limited research time (23).

Since forward genetic dissection of favorable traits is a time-consuming and arduous task in basidiomycete mushrooms, comprehensive analysis using omics technologies has become a feasible option. In this study, five different sporocarp developmental stages (S1–S5) of *G. leucocontextum* and *G. lucidum* were selected, and through the combined transcriptome and metabolome analysis, we aimed to preliminarily clarify the differences in active ingredients and key genes related to active substances in *G. leucocontextum* compared with traditional *Ganoderma* (*G. lucidum*; Chizhi). Particular attention is paid to the pharmacologically active triterpenoids among these compounds, such as ganoderic acids (GAs) and ganoleuconin, also with their corresponding genes.

## RESULTS

### Metabolite profiles during the development of *G. leucocontextum* and *G. lucidum*

**Fig 1:**
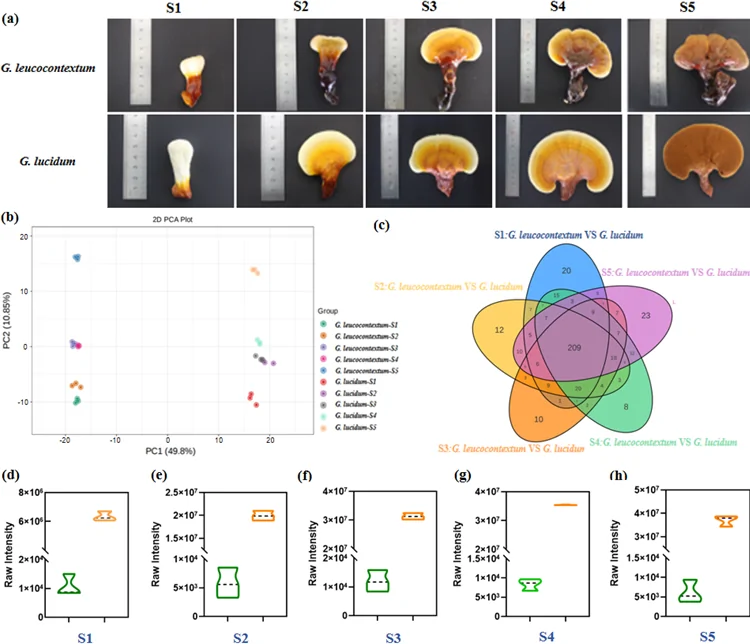
(**a**) The phenotype of the sporocarps of *G. leucocontextum* and *G. lucidum* during five different developmental stages (S1: budding stage; S2: pileus differentiation stage; S3: expansion stage; S4: maturation stage; and S5: spore release stage). (**b**) Principal component analysis of metabolites during different developmental stages of *G. leucocontextum* and *G. lucidum*. (**c**) Venn diagram of metabolite features detected in sporocysts. (**d–h**) Violin plots of ganoleuconin D content in *G. leucocontextum* and *G. lucidum* during the stages from S1 to S5. Yellow: *G. leucocontextum*; Green: *G. lucidum*. The images of “*G. leucocontextum*: S5” and “*G. lucidum*: S5” in this figure are provided as species reference standards, which are consistently used in Fig. 2a to facilitate direct species recognition across complementary experiments.

The ultra-high performance liquid chromatography tandem mass spectrometry (UPLC-MS/MS) was used to qualitatively and quantitatively analyze metabolites from five different developmental stages of *G. leucocontextum* and *G. lucidum* (Fig. 1a). VIP values were calculated for each altered metabolite and set as the cutoff points for all metabolites obtained from UPLC-QTOF-MS analysis. The metabolites that had VIP values ≥ 1 and *P*-value < 0.05 were considered the most relevant ones for the two culture methods. Principal component analysis was used to uncover the internal structure of multiple variables through several principal components. In this study, principal component 1 (PC1) explained 49.8% of the features of the original data set (Fig. 1b). On the scale of developmental time, group *G. leucocontextum* S5 and group *G. lucidum* S5 (spore release stage) were more distantly separated from the first four stages (S1–S4, S1: budding stage; S2: pileus differentiation stage; S3: expansion stage; and S4: maturation stage), which provides direction for our subsequent studies.

**Fig 2:**
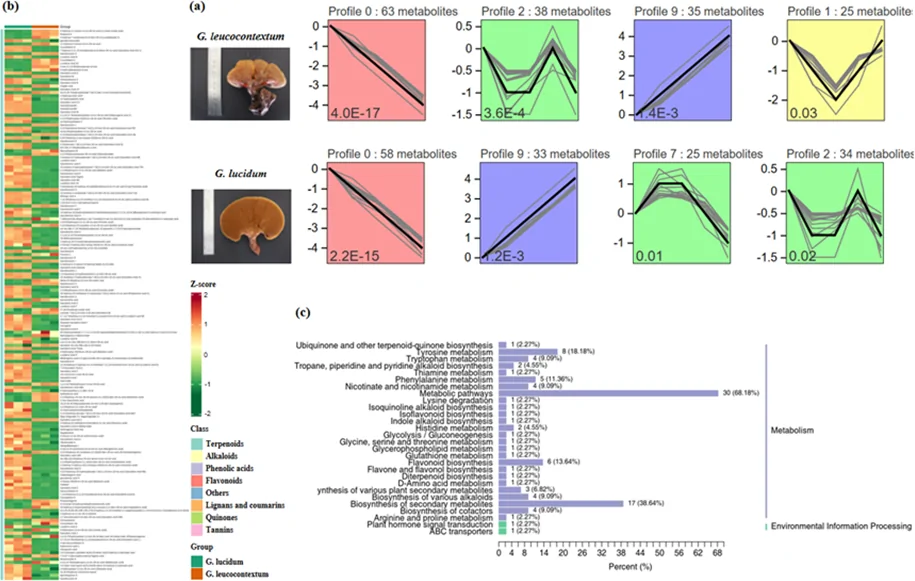
(**a**) Trend analysis of all triterpene substances between *G. leucocontextum* and *G. lucidum*. (**b**) Secondary classification of the main differential metabolites of *G. leucocontextum* and *G. lucidum* during the S5 stage. (**c**) KEGG analysis of major differential metabolites at the S5 stage. The images of “*G. leucocontextum”* and “*G. lucidum”* in panel **a** are identical to those labeled “*G. leucocontextum*: S5” and “*G. lucidum*: S5” in Fig. 1a. This consistent use is intended to unify species visual identification, enabling readers to clearly correlate the data in this figure with the corresponding *Ganoderma* taxa described in Fig. 1.

In total, we ended up with 514 differential metabolites (File S1). The differential metabolites between the two species were 325, 335, 316, 332, and 337 at the stage from S1 to S5 (Fig. 1c). These differential metabolites belong mainly to terpenoids, alkaloids, phenolic acids, flavonoids, lignans and coumarins, quinones, tannins, and steroids; however, steroids are only present from S1 to S4. During the different developmental processes of *Ganoderma*, triterpenoids constituted the primary class of metabolites that differed significantly in abundance between the two species, such as ganoderic acid G, ganoderic acid K, ganoderic acid F, ganoderic acid C1, ganoderic acid GS-2, ganodermic acid D, ganoleuconin L, ganoleuconin J, ganoleuconin D, and ganoleuconin K (Table 1). It is noteworthy that *G. leucocontextum* has consistently higher ganoleucoin than *G. lucidum*; in contrast, nearly all ganoderic acid levels were lower than in *G. lucidum* (Fig. 1d through f). Five identical top 10 differential metabolites have been significantly enriched from S1 to S5, which were ganodermic acid G, ganodermic acid GS-2, ganodermic acid K, ganodermic acid F, and ganoderic acid H. The ganodermic acid GS-2 is also the only ganodermic acid that is found in higher amounts in *G. leucocontextum* than in *G. lucidum*. Subsequent trend analysis of all triterpene substances in *G. leucocontextum* and *G. lucidum* showed that 189 DAMs were distributed across 10 profiles. Among these, profiles 0, 2, 9, and 1 were significantly enriched in *G. leucocontextum*, whereas profiles 0, 9, 7, and 2 were significantly enriched in *G. lucidum*. Profile 1 demonstrated a notable trend for *G. leucocontextum*, characterized by an initial decline and a subsequent rapid rise (Fig. 2). Meanwhile, profile 9 contains most of ganoleuconin, such as ganoleuconin D, ganoleuconin E, and ganoleuconin L.

**TABLE 1:** Differential metabolites of the major triterpenoids between *G. leucocontextum* and *G. lucidum*

Compounds	*Ganoderma* species	S1		S2		S3		S4		S5	
		VIP	*P* value	VIP	*P* value	VIP	*P* value	VIP	*P* value	VIP	*P* value
Ganoderic acid G	*G. lucidum*	1.17E+00	3.12E-05	1.16E+00	3.65E-04	1.15E+00	7.21E-04	1.15E+00	5.45E-04	1.14E+00	2.47E-03
Ganoderic acid K	*G. lucidum*	1.17E+00	5.91E-04	1.16E+00	5.43E-03	1.15E+00	8.74E-04	1.15E+00	2.90E-03	1.14E+00	4.64E-03
Ganoderic acid F	*G. lucidum*	1.16E+00	1.92E-03	1.16E+00	4.22E-04	1.15E+00	4.84E-03	1.15E+00	2.64E-04	1.14E+00	6.89E-04
Ganoderic acid C1	*G. lucidum*	1.17E+00	1.03E-03	1.16E+00	9.83E-04	1.15E+00	1.25E-04	1.15E+00	2.35E-04	1.14E+00	3.79E-04
Ganodermic acid D	*G. lucidum*	1.17E+00	2.28E-04	1.16E+00	4.90E-04	1.15E+00	2.76E-04	1.15E+00	4.68E-05	1.14E+00	1.60E-04
Ganodermic acid GS-2	*G. leucocontextum*	1.17E+00	1.97E-04	1.16E+00	1.24E-04	1.15E+00	5.66E-04	1.15E+00	1.61E-03	1.14E+00	1.72E-04
Ganoleuconin L	*G. leucocontextum*	1.17E+00	1.08E-03	1.16E+00	4.69E-04	1.15E+00	4.69E-04	1.14E+00	1.16E-03	1.14E+00	1.09E-04
Ganoleuconin J	*G. leucocontextum*	1.16E+00	1.32E-04	1.16E+00	1.39E-03	1.15E+00	2.22E-03	1.15E+00	9.06E-04	1.14E+00	3.46E-04
Ganoleuconin D	*G. leucocontextum*	1.16E+00	8.88E-04	1.16E+00	9.30E-04	1.15E+00	4.36E-04	1.15E+00	4.78E-06	1.14E+00	1.34E-03
Ganoleuconin K	*G. leucocontextum*	1.16E+00	8.30E-04	1.16E+00	2.93E-05	1.15E+00	1.27E-03	1.15E+00	8.72E-04	1.14E+00	6.83E-04

During the S5 stage, identified as a critical developmental period, significant differences were observed in the triterpenoid profiles of *G. leucocontextum* and *G. lucidum* (Fig. 2b). These differentially abundant triterpenoids constituted 50.7% of the total differential metabolites. KEGG analysis of differential metabolites revealed that, in addition to “Diterpenoid biosynthesis,” the most highly enriched terms were “Metabolic” and “Biosynthesis of secondary metabolites,” both of which are associated with triterpenoids (Fig. 2c).

### Differential gene expression profiles

**Fig 3:**
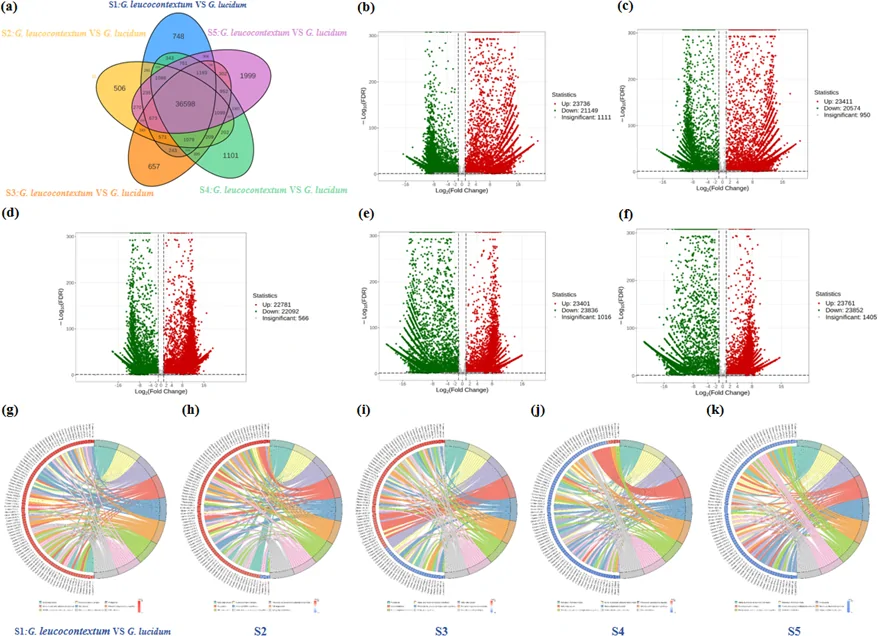
The transcriptome analysis in different developmental stages. (**a**) The Venn diagram of DEGs between *G. leucocontextum* and *G. lucidum* during the same developmental stage. (**b–f**) Volcano maps of upregulated and downregulated genes in *G. leucocontextum* and *G. lucidum* from S1 to S5. (**g–k**) Statistics of KEGG pathway enrichment. The bar plots show cluster IDs obtained by resequencing on the left and pathways enriched for the top nine on the right.

We applied RNA-seq analysis of differential expression at transcript resolution to capture the transcriptome dynamics in *G. leucocontextum* and *G. lucidum* at different development stages. We finally obtained a total of 227.61 Gb clean data, with each library of samples reaching 6 Gb of clean data, and the percentage of *Q*30 bases was above 93% (File S2). A total of 228,593 significantly differentially expressed genes (DEGs) were identified among the five compared combinations. Specifically, there were 44,885, 43,985, 44,873, 47,237, and 47,613 DEGs identified for S1, S2, S3, S4, and S5 between both species (Fig. 3). It can be seen that the number of DEGs did not fluctuate much in these five comparison groups (Fig. 3). The KEGG pathways enriched by the DEGs included valine, leucine, and isoleucine biosynthesis from S1 to S5. In addition to this, there were two other biological pathways that were enriched by four periods, which were the sulfur relay system and phenylalanine, tyrosine, and tryptophan biosynthesis.

To identify the key biological pathways involved in the developmental stages of *Ganoderma*, we conducted KEGG pathway analysis on the respective stages of *G. leucocontextum* and *G. lucidum* (Fig. 3g through k). During the S5 stage, identified as a critical developmental period, significant differences were observed in the triterpenoid profiles of *G. leucocontextum* and *G. lucidum* (Fig. 2b). These differentially abundant triterpenoids constituted 50.7% of the total differential metabolites. We hypothesize that the enrichment of these two pathways is due to the fact that *Ganoderma* development requires intermediate metabolites such as NADH, glyoxylate, and dicarboxylate in the tricarboxylic acid cycle.

In addition, the glycolysis/gluconeogenesis pathway was co-enriched in both *Ganoderma* species. *G. leucocontextum* specifically showed enrichment of the isoflavone biosynthesis pathway at the S2 stage. Three isoflavones detected in the metabolomic data were used for validation, and their levels in S2 were not significantly higher than those in other developmental stages.

**Fig 4:**
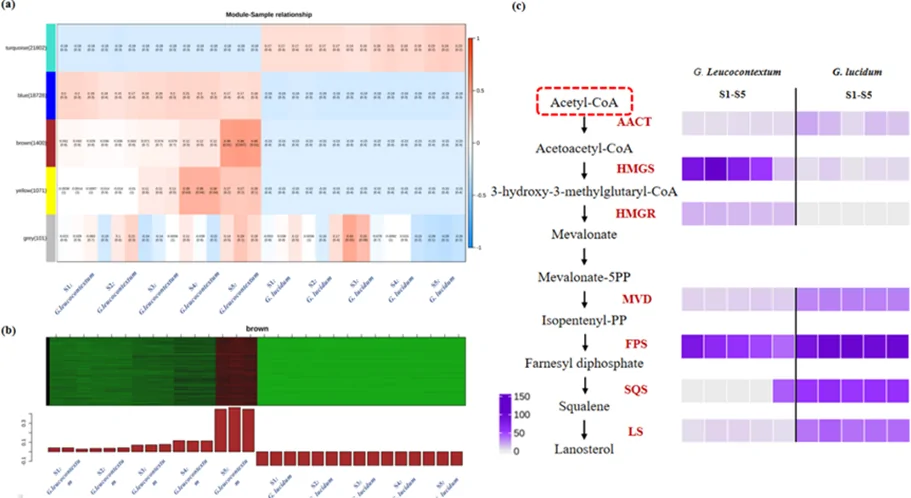
(**a**) The weighted gene co-expression network analysis of DEGs. Clustering dendrogram showing 21 modules of co-expressed genes on the basis of WGCNA. (**b**) Trends in the correlation of 1,400 genes in the brown module with five developmental stages between *G. leucocontextum* and *G. lucidum*. (**c**) Heat map of the expression of genes related to ganoderic acid metabolic pathway from S1 to S5 stages.

The S5 stage was enriched for the valine, leucine, and isoleucine degradation pathway and the flavonoid biosynthesis pathway (Fig. 3k). Weighted Gene Co-expression Network Analysis (WGCNA) identified a brown module highly associated with the S5 stage of *G. leucocontextum*, and this module contained a total of 1,400 genes (Fig. 4a through c).

Ganoderic acid and ergosterol produced by *Ganoderma* exhibit pharmacological activity. The relative expression levels of seven key genes in the triterpenoid biosynthesis pathway are shown in Fig. 4c. The expression of *HMGS* and *HMGR* in *G. leucocontextum* was higher than *G. lucidum* at all five developmental stages. The downstream genes in the triterpenoid synthesis pathway showed higher expression in *G. lucidum*, which included the following: *MVD*, *FPS*, *SQS,* and *LS*. The result is consistent with the metabolomic data. Lanosterol is a precursor substance of ganoderic acid, and higher expression of the downstream genes implies higher ganoderic acid content.

**Fig 5:**
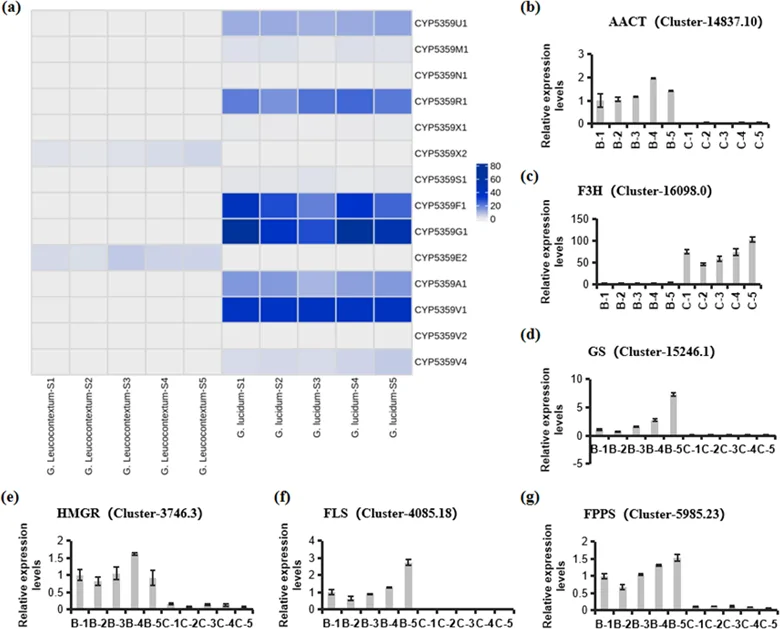
(**a**) Heatmap of the expression of 14 arbitrarily selected genes specifically present in the CYP450 family of genes in *Ganoderma*. (**b–g**) The genes were screened for use in quantitative real-time PCR experiments (which include the key upstream genes *HMGR* and *AACT* and the two downstream CYP450 family genes of the ganoderic acid metabolism pathway, two key flavonoid metabolism pathway genes *FLS* and *F3H*, and the glutamine synthetase gene *GS*).

After the synthesis of lanosterol, the carbon skeleton of the triterpene substance requires the participation of *CYP450* in the modification reaction to form compounds such as GAs. In order to understand the expression pattern of such important genes during the development of *G. leucocontextum* and *G. lucidum*, we arbitrarily selected 14 genes from the largest gene family of *CYP450* genes and heatmapped their expression (Fig. 5a). It can be seen that most of the genes are expressed at higher levels in *G. lucidum*, except for the *CYP5359X2*, *CYP5359E2,* and *CYP5359V2*. Almost all *CYP450* genes of *G. lucidum* peaked in expression during the S1 and S5 stages.

### Transcription factors

A total of 2,786 transcription factor genes were identified through transcriptome data analysis and could be grouped into 50 transcription factor families, the majority of which were “C2H2,” “zn-clus,” “TRAF,” “GNAT,” “C3H,” “HMG,” “SET,” “SNF2,” and “HB-other.” These nine families account for 75.7% of the total number of all predicted transcription factors. Of these 2,786 transcription factor genes, 6 were annotated as being associated with valine, leucine, and isoleucine biosynthesis. “Cluster 17321.2,” “cluster 17321.4,” “cluster 6445.10,” “cluster 6445.13,” “cluster 6445.11,” and “cluster 6445.18” belonged to “HSF” and “GNAT”. These transcription factors may be involved in the biosynthesis of triterpenoid substances (Fig. 5h and Table 2).

**TABLE 2:** Identified transcription factors potentially associated with triterpenoid metabolism

ID	KEGG	TrEMBL	Pfam	TF classification
Cluster 17321.2	ko00280; ko00640; ko01110; ko01100	A0A7S2UTI8_9STRA SubName: Full=Hypothetical protein {ECO:0000313|EMBL:CAD9858802.1}	Transketolase, pyrimidine-binding domain	HSF
Cluster 17321.4	ko00280; ko00640; ko01110; ko01100	A0A7S2UTI8_9STRA SubName: Full=Hypothetical protein {ECO:0000313|EMBL:CAD9858802.1}	Transketolase, pyrimidine-binding domain	HSF
Cluster 6445.10	ko00010; ko00053; ko00071; ko00280; ko01110	A0A836AM02_9ROSI SubName: Full=Aldehyde dehydrogenase (NAD(P)(+)) ald {ECO:0000313|EMBL:KAG5220764.1}	Acetyltransferase (GNAT) domain	GNAT
Cluster 6445.13	ko00010; ko00053; ko00071; ko00280; ko01110	A0A836AM02_9ROSI SubName: Full=Aldehyde dehydrogenase (NAD(P)(+)) ald {ECO:0000313|EMBL:KAG5220764.1}	Acetyltransferase (GNAT) domain	GNAT
Cluster 6445.11	ko00010; ko00053; ko00071; ko00280; ko01110	A0A836AM02_9ROSI SubName: Full=Aldehyde dehydrogenase (NAD(P)(+)) ald {ECO:0000313|EMBL:KAG5220764.1}	Acetyltransferase (GNAT) domain	GNAT

To validate the RNA-seq results, we selected six DEGs (which include the key upstream genes *HMGR* and *AACT* and the two downstream *CYP450* family genes of the ganoderic acid metabolism pathway, two key flavonoid metabolism pathway genes *FLS* and *F3H*, and the glutamine synthetase gene *GS*) and analyzed their expression levels from S1 to S5 using RT-qPCR. The expression trends of these genes were largely consistent with transcriptomic data. Among them, the heatmap of transcriptome expression of *HMGR* and *AACT* has been demonstrated in Fig. 4a. The results validated the relevance of RNA-seq data, and RT-qPCR showed good consistency in gene expression trends (Fig. 5b through g).

## DISCUSSION

Plant secondary metabolites have important uses in the food and pharmaceutical industries, such as fine chemicals and cosmetics. The biosynthesis, regulation, and metabolic engineering of useful secondary metabolites have been extensively studied (24). More than 400 secondary metabolites have been isolated from *Ganoderma* through phytochemical studies. The major isolates were lanosterane-type triterpenes (ganoderic acid and lucidenic acid), meroterpenes, steroids, and their various derivatives (25). In this study, through metabolome analysis on two different *Ganoderma* at five developmental stages, a total of 663 metabolites were obtained. The content of most ganoderic acids is consistently higher in *G. lucidum* than in *G. leucocontextum*, which includes ganoderic acid G, ganoderic acid F, and ganoderic acid H. Ganoderic acids, highly oxygenated C30 lanostane-type triterpenoids, are the prominent bioactive constituents in the genus *Ganoderma*. The high content of ganoderic acid is not unexpected in *G. lucidum* as a traditional medicinal fungus. The biosynthesis of terpenoids involves two pathways: the mevalonate (MVA) pathway and the pyruvate/glyceraldehyde-3-phosphate pathway. After adding ^13^C-labeled compounds to the liquid medium of *G. lucidum* in the ^13^C isotope-labeling experiment, ^13^C-labeled 3α and 3β triterpenoids were detected in the metabolites. This indicates that *Ganoderma* triterpenoids are synthesized via the MVA pathway. Therefore, we focused on the key genes in the triterpenoid biosynthesis pathway of the MVA pathway in *Ganoderma*. In the early stages of triterpenoid biosynthesis (from acetyl-CoA to acetoacetyl-CoA and mevalonate), there was no significant difference in gene expression between the two species. However, from mevalonate-5-diphosphate to isopentenyl pyrophosphate, and until the synthesis of lanosterol, which is the precursor of most ganoderic acids, the expression levels of key genes in *G. lucidum* were consistently significantly higher than those in *G. leucocontextum*. This result is consistent with the metabolomics data. The higher content of ganoderic acids in *G. leucocontextum* is attributed to the high expression of downstream genes in the triterpenoid biosynthesis pathway.

Cytochrome P450 monooxygenases (CYP450s) play a key role in both the oxidation of xenobiotic compounds and in fungal primary and secondary metabolism. For example, in *Ganoderma,* CYP450 is responsible for catalyzing the conversion of lanosterol to ganoderic acid (26, 27). By comparing the transcriptomic and metabolomic differences of Ganoderma in liquid superficial-static culture and submerged culture, it was revealed that the *CYP5150L8* was the key gene regulating lanosterol flux into ganoderic acid biosynthesis (28). A previous study reported the identification, annotation, and phylogenetic classification of P450s in Polyporales, *Bjerkandera adusta*, *Ganoderma*, and *Phlebia brevispora*. The results indicate that the *Ganoderma lucidum* (10597 SS1) genome contains a total of 209 *CYP450* genes, including 41 gene families, of which *CYP5359* is the largest, and that this gene family is specifically present in *Ganoderma lucidum*. Multiple P450s belonging to different *CYP* families were found in the vicinity of secondary metabolic pathway genes, suggesting that they are involved in the biosynthesis of secondary metabolites (29). Therefore, we arbitrarily selected 14 genes in the *CYP5359* family to understand their expression patterns in different *Ganoderma* species. The high expression of most *CYP5359* in *Ganoderma lucidum* is consistent with the higher GA content of *Ganoderma lucidum* in the metabolomic data. In another study, 78 *CYP450* genes were upregulated and expressed in the mycelium of *Ganoderma lucidum* for prophylactic purposes, and changes in the expression of these genes were positively correlated with changes in triterpene content. The researchers concluded that these 78 *CYP450s* were associated with the biosynthesis of triterpenes in *Ganoderma lucidum* (30). Member *CYP515018* of P450s has been implicated for a similar role in the biosynthesis of secondary metabolites such as triterpenoids (31). Based on these results, the specificity and quantitative dominance of the CYP5359 family in the *Ganoderma* genome strongly suggest its involvement in triterpene biosynthesis. Therefore, this gene family is a primary target for subsequent investigation, although experimental validation is required to confirm this hypothesis.

Transcription factors play a central role in orchestrating spatiotemporally precise gene expression programs that are essential for proper control of growth and development in all organisms (32–34). In this study, transcriptome analysis identified six transcription factors from the HSF and GNAT families that are putatively involved in triterpene synthesis. The biosynthesis of triterpenes in *Ganoderma* begins with acetyl coenzyme A and synthesizes the triterpene precursor substance lanosterol via the mevalonate pathway (35, 36). Acetylation modification is one of the major modifications of intracellular proteins, and the acetyl group is usually in the form of acetyl coenzyme A or acetyl phosphate (37). The GNAT family is a relatively comprehensive class of acetyltransferases known so far (38). Therefore, we hypothesized that the two GNAT transcription factors may affect the synthesis of *Ganoderma* triterpenoids by regulating the acetylation process of *Ganoderma lucidum*, which in turn affects its internal acetyl coenzyme A content. In response to heat stress (HS), plants accumulate a large amount of heat shock protein (HSPs), which mainly acts as a molecular chaperone to prevent protein aggregation and promote the refolding of heat-damaged proteins, so as to achieve effective high temperature defense; in response to high temperature stress, the expression of heat shock protein is mainly regulated by HSFs (heat stress transcription factors) (39). Many studies have demonstrated that HS treatment of *Ganoderma* induces the accumulation of HSPs and enhances the biosynthesis of GAs (40 ). In addition, ROS and Ca^2+^ have been shown to be involved in the regulation of GA biosynthesis by HS (40). After Ca^2+^ was added to the static liquid fermentation medium of *Ganoderma*, the total GA yield was increased by 3.7 times, ganoderic acid MK, ganoderic acid T, ganoderic acid S, and ganoderic acid Me yield was increased by 2.6–4.5 times, and the expression of three Ca^2+^ sensor genes (*cam*, *can,* and *crz1*) and three triterpenoid biosynthesis key genes (*Gl-hmgr*, *Gl-sas,* and *Gl-ls*) was significantly upregulated (41, 42). Silencing *GPx*, one of the most important enzymes that directly regulates ROS in *Ganoderma*, will result in a decrease in GA content, which can be restored by the addition of exogenous Ca^2+^, suggesting a correlation between ROS and Ca^2+^ in the regulation of *Ganoderma* triterpene biosynthesis (43). Indeed, it was found through studies in *Arabidopsis* and wheat that Ca^2+^ can directly regulate the transcriptional activity of HSF (44, 45). ROS can either directly affect HSF oligomerization or lead to further HSF activation through the MAPK pathway (46). In summary, HSF can affect the triterpenoid content of *Ganoderma* through three aspects: heat stress, Ca^2+^, and ROS.

## MATERIALS AND METHODS

### Sample collection at differential stages

*G. leucocontextum* and *G. lucidum* were provided by the Vegetable Research Institute of Tibet Autonomous Region Academy of Agricultural and Animal Husbandry Sciences of China. After 20 days of growth on sorghum medium at 25°C, mycelia were inoculated onto the substrate packed in heat-sealed cultivation bags with microfilter windows and cultured in the dark at 25°C for 1 month. The sorghum-based medium was specifically designed to provide optimal nutritional supplementation for *Ganoderma* growth, while concurrently establishing a porous matrix structure with superior moisture retention properties. For fruiting body growth, the bags were cultured in a room with 10 h of illumination and 30 min of ventilation at 26°C. In order to study the differences in transcriptional and metabolic pathways during different developmental processes between *G. leucocontextum* and *G. lucidum*, we selected five important developmental stages (S1: budding stage; S2: pileus differentiation stage; S3: expansion stage; S4: maturation stage; and S5: spore release stage) of the two species as test materials. For consistency in species identification, the reference images of *G. leucocontextum* and *G. lucidum* used in Fig. 2a are the same as those in Fig. 1a (labeled “*G. leucocontextum*: S5” and “*G. lucidum*: S5”). This design ensures that readers can accurately associate experimental data across different figures with the target fungal species, avoiding potential confusion from variable species representation.

### RNA sequencing

Abaxial sides of the pileus from five independent bags with the same developmental stage were selected and frozen in liquid nitrogen. Three replicate samples were prepared for each developmental stage. All samples were maintained at −80°C until subsequent RNA extraction. RNA extraction, cDNA library construction, and sequencing-ready library preparation were conducted, followed by high-throughput sequencing performed by Biomarker Technologies Co., Ltd. (Beijing, China, http://www.biomarker.com.cn/). Transcript abundances were presented as normalized fragments per kilobase of transcript per million mapped reads. On the basis of the model of negative binomial distribution, DESeq R package (1.12.1) was used to perform differential expression analysis by providing statistical routines used to determine differential expression by means of the digital data of gene expression. Genes demonstrating a fold change > 2 with a statistically significant threshold (*P* < 0.05) were classified as differentially expressed between comparative groups. Transcription factor prediction uses iTAK software, which integrates two databases, plnTFDB and PlantTFDB.

### Quantitative real-time PCR validation

The cDNA used for quantitative real-time PCR came from the same batch of samples that were sequenced and returned by Biomarker Technologies. The LightCycler 480 real-time PCR system with a 96-well plate was used to conduct amplification reaction at 95°C for 5 min, followed by 45 cycles of 10 s at 95°C, 20 s at 60°C, and 20 s at 72°C in a volume of 10 µL. At the end of each experiment, a melt-curve analysis was carried out using the default parameters (5 s at 95°C and 1 min at 65°C). Actinidia β-actin was used for normalization. All analyses were repeated three times using biological replicates. The relative expressions were calculated using the 2 −∆∆Ct method.

### Extensive metabolomics analysis

The test material used for metabolome analysis in this study was identical to that used for transcriptome sequencing. Three replicate samples were prepared for each developmental stage, and the two species contain a total of 30 samples. The metabolic group of this research project is based on the UPLC-MC/MS monitoring platform. Based on Biomarker’s own database, metabolite characterization was performed based on secondary spectral information, and duplicate signals containing K^+^, Na^+^, and NH4^+^ were removed at the same time. Metabolite quantification was accomplished using multiple reaction monitoring mode analysis by triple quadrupole mass spectrometry. Mass spectrometry data were processed using Analyst software (version 1.6.3). Quality control samples, prepared by pooling aliquots of sample extracts, were employed to evaluate analytical reproducibility under identical experimental conditions.

## Supplementary Material

Reviewer comments

## Data Availability

The transcriptomic data have been deposited in the National Center for Biotechnology Information (NCBI) database under the accession number PRJNA1417288. The metabolomic data have been deposited in the MetaboLights database under the accession number MTBLS13871. All data necessary to validate the conclusions of this study are accessible through the aforementioned channels.

## SUPPLEMENTAL MATERIAL

File S1 spectrum.01686-25-s0001.xlsx All metabolism from S1 to S5.

File S2 spectrum.01686-25-s0002.xlsx Transcriptome sequencing information for all samples.

OPEN PEER REVIEW reviewer-comments.pdf An accounting of the reviewer comments and feedback.

ASM does not own the copyrights to Supplemental Material that may be linked to, or accessed through, an article. The authors have granted ASM a non-exclusive, world-wide license to publish the Supplemental Material files. Please contact the corresponding author directly for reuse.
