# Supplementary material for: Integrative analysis of metabolome and transcriptome reveals new insights into the formation and regulation of major components of G. leucocontextum and G. lucidum
Source: Microbiol Spectr. 2026 May 18;14(7):e01686-25. doi: 10.1128/spectrum.01686-25 (PMC13340093; doi:10.1128/spectrum.01686-25)
Supplement: Reviewer comments [file reviewer-comments.pdf]

AMERICAN SOCIETY FOR MICROBIOLOGY | Microbiology Spectrum

Integrative analysis of metabolome and transcriptome reveals new insights into formation and regulation of major components of *leucocontextum*and *G. lucidum*

Huijuan Sun, Xuefeng Zhu, Junli Zhang, Lei Gao, Gason Robeb, and Yuanzuo Lv

Corresponding Author(s): Yuanzuo Lv, Hebei Normal University of Science and Technology

Review Timeline:

Submission Date:	June 4, 2025
Editorial Decision:	August 7, 2025
Revision Received:	November 14, 2025
Accepted:	November 22, 2025

Editor: Yuheng Yang

Reviewer(s): Disclosure of reviewer identity is with reference to reviewer comments included in decision letter(s). The following individuals involved in review of your submission have agreed to reveal their identity: Aisha aish (Reviewer #2)

Transaction Report:

(Note: With the exception of the correction of typographical or spelling errors that could be a source of ambiguity, letters and reports are not edited. The original formatting of letters and referee reports may not be reflected in this compilation.)

DOI: https://doi.org/10.1128/spectrum.01686-25

Re: Spectrum01686-25 (**Integrative analysis of metabolome and transcriptome reveals new insights into formation and regulation of major components of *leucocontextum*and *G. lucidum***)

Dear Dr. Huijuan Sun:

Thank you for the privilege of reviewing your work. Below you will find my comments, instructions from the Spectrum editorial office, and the reviewer comments.

Please return the manuscript within 60 days; if you cannot complete the modification within this time period, please contact me. If you do not wish to modify the manuscript and prefer to submit it to another journal, notify me immediately so that the manuscript may be formally withdrawn from consideration by Spectrum.

Revision Guidelines
To submit your modified manuscript, log into the submission site at https://spectrum.msubmit.net/cgi-bin/main.plex. Go to Author Tasks and click the appropriate manuscript title to begin. The information you entered when you first submitted the paper will be displayed; update this as necessary. Note the following requirements:

• Upload point-by-point responses to the issues raised by the reviewers in a file named "Response to Reviewers," NOT in your cover letter.
• Upload a compare copy of the manuscript (without figures) as a "Marked-Up Manuscript" file.
• Upload a clean .DOC/.DOCX version of the revised manuscript and remove the previous version.
• Each figure must be uploaded as a separate, editable, high-resolution file (TIFF or EPS preferred), and any multipanel figures must be assembled into one file.
• Any supplemental material intended for posting by ASM should be uploaded with their legends separate from the main manuscript. You can combine all supplemental material into one file (preferred) or split it into a maximum of 10 files with all associated legends included.

For complete guidelines on revision requirements, see our Submission and Review Process webpage. Submission of a paper that does not conform to guidelines may delay acceptance of your manuscript.

Data availability: ASM policy requires that data be available to the public upon online posting of the article, so please verify all links to sequence records, if present, and make sure that each number retrieves the full record of the data. If a new accession number is not linked or a link is broken, provide Spectrum production staff with the correct URL for the record. If the accession numbers for new data are not publicly accessible before the expected online posting of the article, publication may be delayed; please contact production staff (Spectrum@asmusa.org) immediately with the expected release date.

Publication Fees: For information on publication fees and which article types are subject to charges, visit ourwebsite. If your manuscript is accepted for publication and any fees apply, you will be contacted separately about payment during the production process; please follow the instructions in that e-mail. Arrangements for payment must be made before your article is published.

ASM Membership: Corresponding authors may join or renew ASM membership to obtain discounts on publication fees. Need to upgrade your membership level? Please contact Customer Service at Service@asmusa.org.

The ASM Journals program strives for constant improvement in our submission and publication process. Please tell us how we can improve your experience by taking this quick Author Survey.

Thank you for submitting your paper to Spectrum.

Sincerely,
Yuheng Yang
Editor
Microbiology Spectrum

Reviewer #1 (Comments for the Author):

This study provides a timely and important investigation of two Ganoderma species, the newly discovered G. leucocontextum and the traditional G. lucidum. This comparative approach has significant scientific value and fills the knowledge gaps clearly identified in the introduction. The approach is robust and comprehensive by combining ultra-performance liquid chromatography tandem mass spectrometry (UPLC-MS/MS) metabolomics and RNA sequencing (RNA-seq) transcriptomics technologies to

perform integrated omics analysis at multiple developmental stages. This multi-omics strategy is essential for uncovering complex biological pathways and their regulatory mechanisms.

The quality of the RNA-seq raw data was extremely high, with a total clean data volume of 227.61 Gb, a clean data volume of 6 Gb per library, and a Q30 base percentage of more than 93%. This indicates that the experimental execution was performed to a high standard and laid the foundation for the reliability of the transcriptomic findings. In addition, the validation of the RNA-seq results for selected key genes by RT-qPCR significantly enhanced the reliability of the gene expression findings. This demonstrates a commitment to data integrity and increases confidence in the reported gene expression patterns.

The key findings of the study, such as the differential accumulation of mannitol in Ganoderma lucidum, the differential accumulation of ganoderic acid in Ganoderma lucidum, and the identification of potential regulatory genes (such as CYP450s) and transcription factors (HSF and GNAT families), represent a major contribution to Ganoderma lucidum research. These findings provide new insights into species-specific metabolic pathways.

Despite the many strengths of the study, the manuscript has significant room for improvement in the following areas:

First, the manuscript contains numerous awkward wording, grammatical errors, and unnatural expressions that seriously affect its professional presentation. For submission to a high-impact journal such as Microbiology Spectra, clarity and conciseness of language are essential.

Second, the Results section occasionally contains explanatory or hypothetical statements that are better placed in the Discussion section. A strict distinction between these two sections would help improve the objectivity of the results presentation and improve the logical flow.

Third, the depth of the Discussion and causal inferences could be improved. While the Discussion integrates the study findings with the existing literature, it could be further deepened in terms of mechanistic explanations and causal links between the observed gene expression changes and metabolite profiles. Some hypotheses are proposed without sufficient support from direct evidence from the study itself, and it is necessary to clarify more clearly which are observations and which are inferences.

Finally, the quality, labeling, and overall aesthetics of the figures and tables need to be improved to meet the journal standards. There may be specific issues with the legend, the type of figure (e.g., "ring plot" vs. bar chart), or the data presentation that affect immediate understanding.

Reviewer #2 (Comments for the Author):

The detailed suggestions are mentioned in the attached annotated manuscript. Kindly carefully address all the suggestions as scientific names are neglected the standard format.

1. The manuscript generally follows the standard IMRaD (Introduction, Methods, Results, Discussion) structure. This is the generally accepted and most effective structure for scientific manuscripts, which helps improve clarity, logical flow, and reader comprehension. The distinction between the Results and Discussion sections is blurred, with explanatory statements appearing in the Results section, which needs to be addressed. It is recommended to ensure a clear and consistent distinction between the objective presentation of experimental observations in the Results section and the explanatory analysis, hypothesis generation, and connections to existing literature in the Discussion section. This structural improvement will significantly improve the logical flow, scientific integrity, and overall readability of the manuscript, bringing it into line with standard journal publishing practices.

2. Recommended to be added a new table (Table 2. Summary of key differentially expressed genes (DEGs) in the triterpenoid biosynthesis pathway) in the manuscript, which can directly links the transcriptomic findings with the metabolomic results. By summarizing the expression levels of genes known to be involved in the triterpenoid biosynthesis pathway, it provides a molecular basis for the observed differences in metabolite accumulation.

3. Recommended to be added a new table (Table 3. Identified transcription factors that may be involved in triterpenoid metabolism) in the manuscript. The identification of transcription factors is an important new finding of this study. A dedicated table will clearly present these key regulatory elements in a concise and organized manner, making their identification prominent. Transcription factors are major regulators of gene expression and are the main targets for metabolic engineering or functional validation studies. Their clear listing provides a direct roadmap for future studies aimed at validating their precise role and manipulating their expression to enhance triterpenoid biosynthesis.

4. There are many inaccurate and unsuccinct language expressions in the manuscript, which need to be carefully revised. The following are specific examples and modification suggestions:

Line 26: “In cantrast, G. lucidum...” → Change “cantrast” to “contrast”.

Line 28: “...belonging two families...” → Change to “...belonging to two families...”

Line 28: “...HSF and GANT.” → Change “GANT” to “GNAT” to be consistent with the subsequent mention (line 164).

Line 31: “Ganoderma belong to the kingdom...” → Change “belong” to “belongs”.

Lines 33-35: “Although Ganoderma fruiting bodies are not classified as edible mushrooms because they are thick and corky and do not have 34 fleshy textural characteristics, they have been widely studied as medicinal mushroom due to their 35 rich content of bioactive components...” → Reword to improve flow and precision. “do not have fleshy textural characteristics” is clumsy. It is recommended to change to: "Although Ganoderma fruiting bodies are not classified as edible mushrooms due

to their thick and corky texture, they have been widely studied as medicinal mushrooms owing to their rich content of bioactive components..."

Lines 39-40: “Ganoderma lucidum to have shown Ganoderma to have a variety of effects...” → The phrase is repeated. It is recommended to change it to: "Ganoderma lucidum has been shown to have a variety of effects..."

Lines 59-62: “Although the pharmacological activities have been demonstrated, the genes and metabolic pathways involved in the crucial stages of G. leucocontextum is still incomplete due to the limited research time (Liu et al., 2020).” → Corrected many errors: “Although the pharmacological activities have been demonstrated, the genes and metabolic pathways involved in the crucial stages of G. leucocontextum are still incomplete due to limited research time...”

Lines 92-93: “It is noteworthy that G. leucocontextum has consistently higher ganoleucoin than G. lucidum, in contrast to nearly all ganoderic acid was lower than G. lucidum (Fig 1d f).” → Corrected grammar: “It is noteworthy that G. leucocontextum has consistently higher ganoleucoin than G. lucidum; in In contrast, nearly all ganoderic acid levels were lower than in G. lucidum (Fig 1d-f).”

Lines 94-96: “Five identical top 10 differential metabolites have been significantly enriched from 1 to 5, which were ganodermic acid G, ganodermic acid GS-2, ganodermic acid K, ganodermic acid F and ganodericacid H.” → Clarify “enriched from 1 to 5” as “across stages S1 to S5”. Corrected "ganoderic acid" to "ganoderic acid".

Lines 96-97: “The ganodermic acid GS-2 is also the only ganodermic acid that is found in higher amounts in G. leucocontextum than that in G. lucidum..” → Delete the redundant “that in”. Remove extra periods.

Lines 101-102: “Profile 1 of G. leucocontextum contains a unique class of decreasing and then increasing trends (Fig 2a).” → Reworded to improve clarity: “Profile 1 of G. leucocontextum exhibits a unique trend, initially decreasing and then increasing (Fig 2a).”

Lines 106-109: “The KEGG analysis of differential metabolites, in addition to "Diterpenoid biosynthesis", showed the highest enrichment terms of "Metabolic" and "Biosynthesis of secondary metabolites" were also associated with triterpenoids (Fig 2c)." → Corrected grammar: "KEGG analysis of differential metabolites revealed that, in addition to "Diterpenoid biosynthesis," the most highly enriched terms were "Metabolic" and "Biosynthesis of secondary metabolites," both of which are associated with triterpenoids (Fig 2c).

Lines 112-114: “We finally obtained a total of 227.61 Gb clean Data, with each library of samples reaching 6 Gb of clean Data, and the percentage of Q30 bases was above 93% (Supplementary material 2).” → Correct capitalization and pluralization: “We finally obtained a total of 227.61 Gb clean data, with each library of samples reaching 6 Gb of clean data, and the percentage of Q30 bases was above 93% (Supplementary material 2). ”

Line 118: “Valin, leucine and isoleucine biosynthesis” → Change “Valin” to “Valine”.

Line 120: “sulfur relay system” → Consider adding “pathway” to improve clarity,

e.g.: “sulfur relay system pathway”.
Lines 136-139: “Valine decarboxylation allows the production of the precursor substance of triterpenes, acetyl coenzyme A, an important intermediate product of the TCA cycle; and flavonoid may be associated with the pigments accumulated in G. leucocontextum.” → This is a lengthy sentence and contains explanatory statements in the Results section. It is recommended to split and move the explanatory content. It is recommended to change it to: “Valine decarboxylation leads to the production of acetyl coenzyme A, a precursor substance of triterpenes and an important intermediate product of the TCA cycle. Flavonoids, on the other hand, may be associated with the pigments accumulated in G. leucocontextum.” (Move the second sentence to the Discussion section).
Line 144: “7 key genes which in the triterpenoid biosynthesis pathway” → Change it to “7 key genes in the triterpenoid biosynthesis pathway”.
Lines 145-146: “The expression of HMGS and HMGR in G. leucocontextum was higher than that in G. lucidum at all five developmental stages.” → Delete the redundant “that in”.
Lines 146-147: “The genes of downstream in the triterpenoid synthesis pathway...” → Change to “The downstream genes in the triterpenoid synthesis pathway...”
Lines 154-155: “...most of the genes are expressed in G. lucidum, except for the CYP5359X2, CYP5359E2 and CYP5359V2.” → Clarify “expressed”. Is it “higher expression” or “mainly expressed”?
Lines 201-202: “The high expression of most CYP5359 in Ganoderma lucidum equisetum is consistent...” → Correct “equisetum” to “lucidum”.
Lines 207-209: “Member CYP515018 of P450s the have been implicated for a similar role in biosynthesis of second

5. The figures and tables in this manuscript could be improved in terms of clarity, labeling, and overall presentation.
Line 315: “Fig1 (a) The phenotype of the sporocysts...” → Change “sporocysts” to “sporocarps” to be consistent with the text (lines 23, 63).
Lines 319-320: “Violin box plots of ganoleuconin D content...” → The figure shows violin plots, not “violin box plots”. Please correct the description. Also, please ensure that the legend (1d-h) is consistent with the reference to the mannitol/ganoderic acid differences in the text (1d-f) (line 93).
Line 325 (Table 1 title): “Componds” → Change to “Compounds”.
Line 342: “The circos plot shows cluster IDs...” (Legend of Fig. 3g-k) → The figure is a set of bars, not a circumplex. Please fix the descriptions in the figure legends.
General advice: Ensure that all figures have clear, self-explanatory legends that can be understood without extensive reference to the text. Check font size, axis labels, and resolution to ensure optimal readability in publication format. Ensure that species names (e.g., G. leucocontextum, G. lucidum) are always italicized.

6. The manuscript contains inconsistencies in citation format (e.g., "Shiao MS 2003" vs. "Boh et al., 2007" vs. "Li X et al., 2019" vs. "PAN Jun et al., 2021" vs. "Syed, K., Nelson, D. R., Riley, R., et al, 2013").
Recommendations: Strictly follow the specific author guidelines of Microbiology Spectra to standardize the format of all in-text citations and reference lists. Please carefully check the author names, year, journal abbreviations, volume numbers, and page numbers. Thoroughly check whether there are any missing references (cited in the text but not listed) or redundant references (listed but not cited in the text). For example, "Xu YN & Zhong JJ, 2012" in line 240 should be formatted consistently with other references.

Integrative analysis of metabolome and transcriptome reveals new insights into formation and regulation of major components of *G. leucocontextum* and *G. lucidum*

Huijuan Sun[a,*], Xuefeng Zhu[a], Junli Zhang[a], Lei Gao[a], Gason Robeb[b], Yuanzuo Lv[c]
[a]Vegetable Research Institute of Tibet Autonomous Region Academy of Agricultural and Animal Husbandry Sciencess, Lhasa, Tibet, 85000, China
[b]Bomi County Garonggou Village Forest Resources Cultivation Farmers and Herders Specialized Cooperative Society, Lhasa, Tibet, 85000, China
[c]College of Horticulture Science and Technology, Hebei Normal University of Science and Technology, Qinhuangdao, Hebei 066004, China

Corresponding Author：

Huijuan Sun*

Address：Vegetable Research Institute of Tibet Autonomous Region Academy of Agricultural and Animal Husbandry Sciences, Lhasa, Tibet, 85000, China
E-mail：m18898002012@163.com

Abstract: *Ganoderma* is a class of medicinal fungi with diverse medicinal effects, of which triterpenoids are one of the main pharmacologically active substances. In this study, five sporocarps at different developmental stages from two *Ganoderma* species were used as test material for joint transcriptomic and metabolomic analyses. As a newly discovered species, *G. leucocontextum* contains higher levels of ganoleucoin during development. In cantrast, *G. lucidum* consistently contained higher levels of ganoderic acid. Most genes in the triterpenoid synthesis pathway were more highly expressed in *G. lucidum*, including several CYP450 family genes specific to *Ganoderma*. Finally, we identified six putative transcription factors potrntially involved in *Ganoderma* triterpene metabolism, belonging two families, HSF and GANT. Our findings provide two datasets on dynamic gene expression and differential metabolites during the development of different *Ganoderma* species.

Keywords： *G. leucocontextum*; *G. lucidum*; transcriptome; metabolome; ganoleucoin

Introduction

Ganoderma belong to the kingdom of Fungi, division of Basidiomycata, class of Homobasidiomycetes, order of Aphyllophorales, family of Polyporaceae (*Ganodermataceae*) and genus of *Ganoderma* (Richter et al., 2015; Seo and Kirk, 2000). Although *Ganoderma* fruiting bodies are not classified as edible mushrooms because they are thick and corky and do not have fleshy textural characteristics, they have been widely studied as medicinal mushroom due to their rich content of bioactive components including triterpenes, polysaccharides, nucleosides and

alkaloids (Jonathan et al., 2008; Soccol et al., 2016; Xie et al., 2020). In Traditional Chinese Medicine (TCM), *Ganoderma* is described as having the ability to tonify the "qi" and calm the spirit, and to relieve coughs and asthma. Modern pharmacological studies have shown Ganoderma lucidum to have shown *Ganoderma* to have a variety of effects including anti-tumor, anti-hypertensive, anti-viral and immunomodulatory (Boh et al., 2007). These medical functions are mainly derived from a variety of biologically active substances including triterpenoids (Shiao MS 2003). Triterpenoids have a wide range of pharmacological activities such as anti-tumor, hepatoprotective and detoxification, anti-HIV virus and cholesterol lowering (Gao et al., 2003; Min et al., 1998; Komoda et al., 1989). Not only that, but in recent years more and more studies have shown that *Ganoderma* has medical potential in the fight against cancer as well (Li X et al., 2019). The intense bitterness in *Ganoderma* comes from the triterpene and it depends on the strain, cultivation conditions and manufacturing processes (Seo et al., 2009).

According to the international fungal name database Species Fungorum (Index Fungorum Home Page, accessed 19th Dec, 2023), there are 421 accepted species in the genus Ganoderma. There are many species of *Ganoderma*, but not all of them have medicinal value. In fact, the Pharmacopoeia of the Peoples Republic of China (Part 1) (2000, 2005, 2010, and 2015 editions) listed the fruiting body of *Ganoderma lucidum* Karst (Chi Zhi) and *Ganoderma* sinensis as the legal traditional Chinese medicines. *Ganoderma leucocontextum* is a new species discovered in the Linzhi of Tibet, China in 2015 (Li TH et al., 2015). In recent years, new compounds with pharmacological activities such as triterpenes have been isolated from *G. leucocontextum* (PAN Jun et al., 2021; Liu et al., 2018; Wang et al., 2017). It has been suggested that *Ganoderma leucocontextum* contains special triterpenoids and polysaccharide components not found in traditional varieties of *G. lucidum*, and that it has the ability to regulate immune activity and other effects. Although the pharmacological have been demonstrated, the gens and metabbplic pathways involved in the crucial stages of *G. leucocontextum* is still incomplete due to the limited research time (Liu et al., 2020).

Since forward genetic dissection of favorable traits is a time-consuming and arduous task in basidiomycete mushrooms, comprehensive analysis using omics technologies has become a feasible option. In this study, five different sporocarp developmental stages (S1~S5) of *G. leucocontextum* and *G. lucidum* were selected respectively, and through the combined

transcriptome and metabolome analysis, we aimed to preliminarily clarify the differences in active ingredients and key genes related to active substances in *G. leucocontextum* as compared with traditional *Ganoderma* (*G. lucidum*; Chizhi).

Results

Metabolite profiles during development of *G. leucocontextum* and *G. lucidum*

The Ultra-High Performance Liquid Chromatography Tandem Mass Spectrometry (UPLC-MS/MS) was used to quality and quantitatively analyze metabolites from five different developmental stages of *G. leucocontextum* and *G. lucidum* (Fig 1a). VIP values were calculated for each altered metabolite and set as the cut-off points for all metabolites obtained from UPLC-QTOF-MS analysis. The metabolites that had VIP values ≥1 and P-value < 0.05 were considered the most relevant ones for the two culture methods. PCA was used to uncover the internal structure of multiple variables through several principal components. In this study, the principal component 1 (PC1) explains 49.8% of the features of the original dataset (Fig 1b). From the PC1, it can be found that the separation of *G. leucocontextum* and *G. lucidum* occurs due to the different species. The different developmental stage also occurs in the principal component 2 (PC2), which explains 10.85% of the dataset. On the scale of developmental time, group *G. leucocontextum* S5 and group *G. lucidum* S5 were more distantly separated from the first 4 stages (S1~S4), which provides direction for our subsequent studies.

In total we ended up with 514 differential metabolites (Supplementary material 1). The differential metabolites between the two species were 325, 335, 316, 332 and 337 at the stage from S1 to S5 (Fig 1c). These differential metabolites belong mainly to terpenoids, alkaloids, phenolic acids, flavonoids, lignans and coumarins, quinones, tannins and steroids, and however, steroids are only present from S1 to S4. During the different developmental processes of *Ganoderma*, both species significantly different metabolites were mainly enriched in triterpenoids, such as ganoderic acid G、ganoderic acid K、ganoderic acid F、ganoderic acid C1、ganoderic acid GS-2、ganodermic acid D、ganoleuconin L、ganoleuconin J、ganoleuconin D and ganoleuconin K (Table 1). It is worth that *G. leucocontextum* has consistently higher ganoleucoin than *G. lucidum*, in contrast to nearly all ganoderic acid was lower than *G. lucidum*（Fig 1d~f）.

Five identical top 10 differential metabolites have been significantly enriched from S1 to S5, which were ganodermic acid G, ganodermic acid GS-2, ganodermic acid K, ganodermic acid F and ganodericacid H. The ganodermic acid GS-2 is also the only ganodermic acid that is found in higher amounts in *G. leucocontextum* than that in *G. lucidum*.. Subsequent trend analysis of all triterpene substances in *G. leucocontextum* and *G. lucidum* showed that 189 DAMs were distributed across 10 profiles each, with profiles 0, 2, 9 and 1 being significantly enriched between *G. leucocontextum*, and profiles 0, 9, 7 and 2 being significantly enriched in *G. lucidum*. Profile 1 of *G. leucocontextum* contains a unique class of decreasing and then increasing trends (Fig 2a). Meanwhile profile 9 contains most of ganoleuconin, such as ganoleuconin D, ganoleuconin E and ganoleuconin L.

As a potentially critical stage, the Fig. 2b shows the difference in triterpenoids metabolized by *G. leucocontextum* and *G. lucidum* during the S5 stage, which accounted for 50.7% of all differential metabolites. The KEGG analysis of differential metabolites, in addition to "Diterpenoid biosynthesis", showed the highest enrichment terms of "Metabolic" and "Biosynthesis of secondary metabolites" were also associated with triterpenoids (Fig 2c).

Differential gene expression profiles

We applied RNA-seq analysis of differential expression at transcript resolution to capture the transcriptome dynamics in *G. leucocontextum* and *G. lucidum* at different development stages. We finally obtained a total of 227.61 Gb clean Data, with each libraries of samples reaching 6 Gb of clean Data, and the percentage of Q30 bases was above 93% (Supplementary material 2). A total of 228,593 significant differentially expressed genes (DEGs) were identified among the five compared combinations. Specifically, there were 44,885, 43,985, 44,873, 47,237 and 47,613 DEGs were identified for S1, S2, S3, S4 and S5 between both species (Fig 3a). It can be seen that the number of DEGs did not fluctuate much in these five comparison groups (Fig 3b~f). The KEGG pathways enriched by the DEGs included Valin, leucine and isoleucine biosynthesis from S1 to S5. In addition to this there were two other biological pathways that were enriched by four periods which were sulfur relay system and phenylalanine, tyrosine and tryptophan biosynthesis.

Subsequently, in order to identify the main biological pathways involved in the different developmental stages of *Ganoderma*, we performed KEGG analyses on the different

developmental stages of *G. leucocontextum* and *G. lucidum*, respectively (Fig 3g~k). As expected, both species were enriched for biological pathways related to the biosynthesis of triterpenoids such as pyruvate metabolism, terpenoid backbone biosynthesis and ubiquinone and other terpenoid-quinone biosynthesis. In fact, *G. leucocontextum* and *G. lucidum* were also specifically enriched to the Glyoxylate and dicarboxylate and Citrate cycle. We hypothesize that the enrichment of these two pathways is due to the fact that *Ganoderma* development requires intermediate metabolites such as NADH, glyoxylate, and dicarboxylate in the tricarboxylic acid cycle (TCA cycle).

In addition to this, the glycolysis/gluconeogenesis pathway was also co-enriched in both species. This should be related to the fact that they are rich in Ganoderma polysaccharides. *G. leucocontextum* specifically enriched the isoflavone biosynthesis pathway at S2 stage. We used the three isoflavones detected in the metabolomic data for validation and showed that the three isoflavones of S2 were not significantly higher than the other stages.As a potential critical stage, the S5 stage was enriched for valine, leucine and isoleucine degradation pathway and flavonoid biosynthesis pathway (Fig 3k). Valine decarboxylation allows the production of the precursor substance of triterpenes, acetyl coenzyme A, an important intermediate product of the TCA cycle; and flavonoid may be associated with the pigments accumulated in *G. leucocontextum*. WGCNA analysis was used to find a brown module highly associated with the S5 stage of *G. leucocontextum*, and this module contained a total of 1,400 genes (Fig 4a~c).

Ganoderic acid (GAs) and ergosterol produced by *Ganoderma* exhibit pharmacological activity (Zhao RL and He YM, 2018). The relative expression levels of 7 key genes which in the triterpenoid biosynthesis pathway are shown in Fig 4c. The expression of *HMGS* and *HMGR* in *G. leucocontextum* was higher than that in *G. lucidum* at all five developmental stages. The genes of downstream in the triterpenoid synthesis pathway showed higher expression in *G. lucidum*, which included the following *MVD*, *FPS*, *SQS* and *LS*. The result is consistent with the metabolomic data. Lanosterol is a precursor substance of ganoderic acid, and higher expression of the downstream genes implies higher ganoderic acid content.

After the synthesis of lanosterol, the carbon skeleton of the triterpene substance requires the participation of *CYP450* in the modification reaction to form compounds such as GAs. In order to understand the expression pattern of such important genes during the development of *G.*

leucocontextum and *G. lucidum*, we arbitrarily selected 14 genes from the largest gene family of *CYP450* genes and heatmapped their expression (Fig 5a). It can be seen that most of the genes are expressed in *G. lucidum*, except for the *CYP5359X2*, *CYP5359E2* and *CYP5359V2*. Almost all *CYP450* genes of *G. lucidum* peaked in expression during the S1 and S5 stages.

Transcription factors

A total of 2786 transcription factor genes were identified through transcriptome data analysis and could be grouped into 50 transcription factor families, the majority of which were "C2H2", "zn-clus", "TRAF", "GNAT", "C3H", "HMG", "SET", "SNF2" and "HB-other". These nine families account for 75.7% of the total number of all predicted transcription factors. Of these 2786 transcription factor genes, 6 were annotated as being associated with valine, leucine and isoleucine biosynthesis. The "cluster 17321.2", "cluster 17321.4", "cluster 6445.10", "cluster 6445.13", "cluster 6445.11" and "cluster 6445.18" belonged to "HSF" and "GNAT", respectively. These transcription factors may be involved in the biosynthesis of triterpenoid substances (Fig 5h)

To validate the RNA-seq results, we selected 6 DEGs (which include the key upstream genes *HMGR* and *AACT* and the 2 downstream *CYP450* family genes of the ganoderic acid metabolism pathway, 2 key flavonoid metabolism pathway genes *FLS* and *F3H*, and the glutamine synthetase gene *GS*) and analyzed their expression levels from S1 to S5 using RT-qPCR. The expression trends of these genes were largely consistent with transcriptomic data. Among them, the heatmap of transcriptome expression of *HMGR* and *AACT* has been demonstrated in Fig 4. The results validated the relevance of RNA-seq data and RT-qPCR showed good consistency in gene expression trends (Fig5 b~g).

Discussion

Plant secondary metabolites have important uses in the food and pharmaceutical industries, such as fine chemicals and cosmetics. The biosynthesis, regulation and metabolic engineering of useful secondary metabolites have been extensively studied (Zhao J et al., 2005). More than four hundred secondary metabolites have been isolated from *Ganoderma* through phytochemical studies. The major isolates were lanosterane-type triterpenes (ganoderic acid, lucidenic acid), meroterpenes, steroids and their various derivatives. In this study, through metabolome analysis on two different *Ganoderma* at five developmental stages, a total of 663 metabolites were obtained. The content of most ganoderic acids is consistently higher in *G. lucidum* than in *G.*

leucocontextum, which includes ganoderic acid G, ganoderic F, ganoderic H. Ganoderic acids, highly oxygenated C30 lanostane-type triterpenoids, are the prominent bioactive constituents in genus *Ganoderma*. The high content of ganoderic acid is not unexpected in *G. lucidum* as a traditional medicinal fungus.

Cytochrome P450 monooxygenases (CYP450s) played a key role both in xenobiotic compound oxidation and fungai primary and secondary metabolism, for example, in *Ganoderma* CYP450 is reponsible for catalyzing the conversion of lanosterol to ganoderic acid (Syed et al., 2014;Yang et al., 2018). By comparing the transcriptomic and metabolomic differences of Ganoderma in liquid superficial-static culture and submerged culture revealed that the *CYP5150L8* was the key gene regulating lanosterol flux into ganoderic acid biosynthesis (Wang et al., 2020). A previous study reported the identification, annotation and phylogenetic classification of P450ome in Polyporales, *Bjerkandera adusta*, *Ganderma sp.*, *Phlebia brevispora*. The results indicate that the *Ganoderma lucidum* (10597 SS1) genome contains a total of 209 *CYP450* genes, including 41 gene families, of which *CYP5359* is the largest, and that this gene family is specifically present in *Ganoderma lucidum* . Multiple P450s belonging to different *CYP* families were found in the vicinity of secondary metabolic pathway genes, suggesting that they are involved in the biosynthesis of secondary metabolites (Syed K et al., 2013). Therefore, we arbitrarily selected 14 genes in the *CYP5359* family to understand their expression patterns in different *Ganoderma* species. The high expression of most *CYP5359* in *Ganoderma lucidum* equisetum is consistent with the higher GAs content of *Ganoderma lucidum* in the metabolomic data. In another study, 78 *CYP450* genes were up-regulated and expressed in the mycelium of *Ganoderma lucidum* to the prophylactic, and changes in the expression of these genes were positively correlated with changes in triterpene content, and the researchers concluded that these 78 *CYP450s* were associated with the biosynthesis of triterpenes in *Ganoderma lucidum* (Chen et al., 2012). Member CYP5150l8 of P450s the have been implicated for a similar role in biosynthesis of secondary metabolites such as triterpenoids (Wen Wang et al., 2018). Considering these results, we can hypothesize that the specificity and quantitative dominance of the *CYP5359* family in the *Ganoderma* genome suggests a possible relevance to *Ganoderma* triterpene biosynthesis, which is a gene family to focus on in our subsequent studies. Of course, further experimental studies must be designed to address these possibilities.

Transcription factors play a central role in orchestrating spatiotemporally precise gene expression programs that are essential for proper control of growth and development in all organisms (Chu S et al., 1998; Kassir Y et al., 1988; Jing Y et al., 2020). In this study, 6 transcription factors involved in triterpene synthesis, belonging to the HSF and GNAT families, were mined by transcriptome analysis. The biosynthesis of triterpenes in *Ganoderma* begins with acetyl coenzyme A (AcCoA) and synthesizes the triterpene precursor substance lanosterol via the mevalonate (MVA) pathway (Shi L et al., 2010). Acetylation modification is one of the major modifications of intracellular proteins, and the acetyl group is usually in the form of acetyl coenzyme A or acetyl phosphate (AcP) (Hentchel, K. L. & Escalante-Semerena, J. C, 2015). The GNAT family is a relatively comprehensive class of acetyltransferases known so far (Dean X et al., 2015). Therefore, we hypothesized that the two GNAT transcription factors may affect the synthesis of *Ganoderma* triterpenoids by regulating the acetylation process of Ganoderma lucidum, which in turn affects its internal acetyl coenzyme A content. In response to heat stress (HS), plants accumulate a large amount of heat shock protein (HSPs), which mainly acts as a molecular chaperone to prevent protein aggregation and promote the refolding of heat-damaged proteins, so as to achieve effective high temperature defense; in response to high temperature stress, the expression of heat shock protein is mainly regulated by HSFs (heat stress transcription factors) (Huang YC et al., 2016). Many studies have demonstrated that HS treatment of *Ganoderma* induces the accumulation of HSPs and enhances the biosynthesis of GAs (Liu YN et al., 2017; Liu R et al., 2018). In addition, ROS and Ca^{2+} have been shown to be involved in the regulation of GA biosynthesis by HS (Zhang X et al., 2016; Liu YN et al., 2017). When Ca^{2+} was added to the static liquid fermentation medium of *Ganoderma*, the total GA yield was increased by 3.7 times, the ganoderma acid MK, ganoderma acid T, ganoderma acid S and ganoderma acid Me yield was increased by 2.6~4.5 times, and the expression of 3 Ca^{2+} sensor genes (*cam*, *can* and *crz1*) and 3 triterpenoid biosynthesis key genes (*Gl-hmgr*, *Gl-sas* and *Gl-ls*) was significantly up-regulated (Xu YN & Zhong JJ, 2012). Silencing *GPx*, one of the most important enzymes that directly regulates ROS in *Ganoderma*, will result in a decrease in GA content, which can be restored by the addition of exogenous Ca^{2+}, suggesting a correlation between ROS and Ca^{2+} in the regulation of *Ganoderma* triterpene biosynthesis (Li C et al., 2015). Indeed it was found through studies in Arabidopsis and wheat that Ca^{2+} can directly regulate the transcriptional activity of HSF

(Liu HT et al., 2003; Liu HT et al., 2005). ROS can either directly affect HSF oligomerization or lead to further HSF activation through the MAPK pathway (Jacob P et al., 2017). In summary, HSF can affect the triterpenoid content of *Ganoderma* through three aspects: heat stress, Ca^{2+} and ROS.

Materials and Methods

Sample Collection at Differential Stages

G. leucocontextum and *G. lucidum* was provided by Vegetable Research Institute of Tibet Autonomous Region Academy of Agricultural and Animal Husbandry Sciences of China. After 20 days of growth on sorghum medium at 25°C, mycelia were inoculated onto the substrate packed in heat-sealed cultivation bags with microfilter windows and cultured in the dark at 25 ◦C for 1 month. For fruiting body growth, the bags were cultured in a room with 10 h of illumination and 30 min of ventilation at 26 °C. In order to study the differences in transcriptional and metabolic pathways during different developmental processes between *G. leucocontextum* and *G. lucidum*, we selected five important developmental stages (S1: Budding stage; S2: Pileus differentiation stage; S3: Expansion stage; S4: Maturation stage; S5: Spore release stage) of the two species as test materials.

RNA Sequencing

Abaxial sides of the pileus from five independent bags with the same developmental stage were selected and frozen in liquid nitrogen. Three replicate samples were prepared for each developmental stage. All samples were stored at −80°C prior to RNA isolation. RNA isolation, cDNA library construction, sequencing library preparation and high-throughput sequencing were carried out by Biomarker Technologies (Beijing, China, www.biomarker.com.cn). Transcript abundances were presented as normalized fragments per kb of transcript per million mapped reads. On the basic of model of negative binomial distribution, DESeq R package (1.12.1) was used to perform differential expression analysis by providing statistical routines used to determine differential expression by means of the digital data of gene expression. A gene was considered to be significantly differentially expressed if its expression differed between two samples by fold change > 2 and p-value < 0.05. Transcription factor prediction uses iTAK software, which integrates two databases, plnTFDB and PlantTFDB.

Quantitative Real-Time PCR Validation

The cDNA used for qRTPCR came from the same batch of samples that were sequenced and returned by Biomarker Technologies. The LightCycler® 480 real-time PCR system with a 96-well plate was used to conduct an amplified reaction consisting of 95 ℃ for 5 min, followed by 45 cycles of 10 s at 95 ℃, 20 s at 60 ℃, and 20 s at 72 ℃ in a volume of 10 μL. At the end of each experiment, a melt-curve analysis was carried out using the default parameters (5 s at 95℃ and 1 min at 65 ℃). The Actinidia β-actin was used for normalization. All analyses were repeated three times using biological replicates. The relative expressions were calculated using the 2 −ΔΔCt method.

Extensive Metabolomics analysis

The test material used for metabolome analysis in this study was identical to that used for transcriptome sequencing. Three replicate samples were prepared for each developmental stage, the two species contain a total of 30 samples. The metabolic group of this research project is based on the UPLC-MC/MS monitoring platform. Based on the Biomarker's own database, metabolite characterization is performed based on secondary spectral information, and duplicate signals containing K^+, Na^+ and NH4$^+$ are removed at the same time. Metabolite quantification was accomplished using multiple reaction monitoring mode analysis by triple quadrupole mass spectrometry. Mass spectrometry data were processed using Analyst 1.6.3. QC samples were prepared from a mixture of sample extracts and were used to analyze the reproducibility of the samples under the same treatment.

Acknowledgments

This study was jointly funded by the Natural Science Foundation of Tibet Autonomous Region (XZ202101ZR0059G), Tibet Autonomous Region Science and Technology Program (XZ202301ZY0013N) and Tibet Agriculture and Animal Husbandry Science and Technology Innovation Project-Characteristic Understory Resources Development and Utilization Innovation Team.

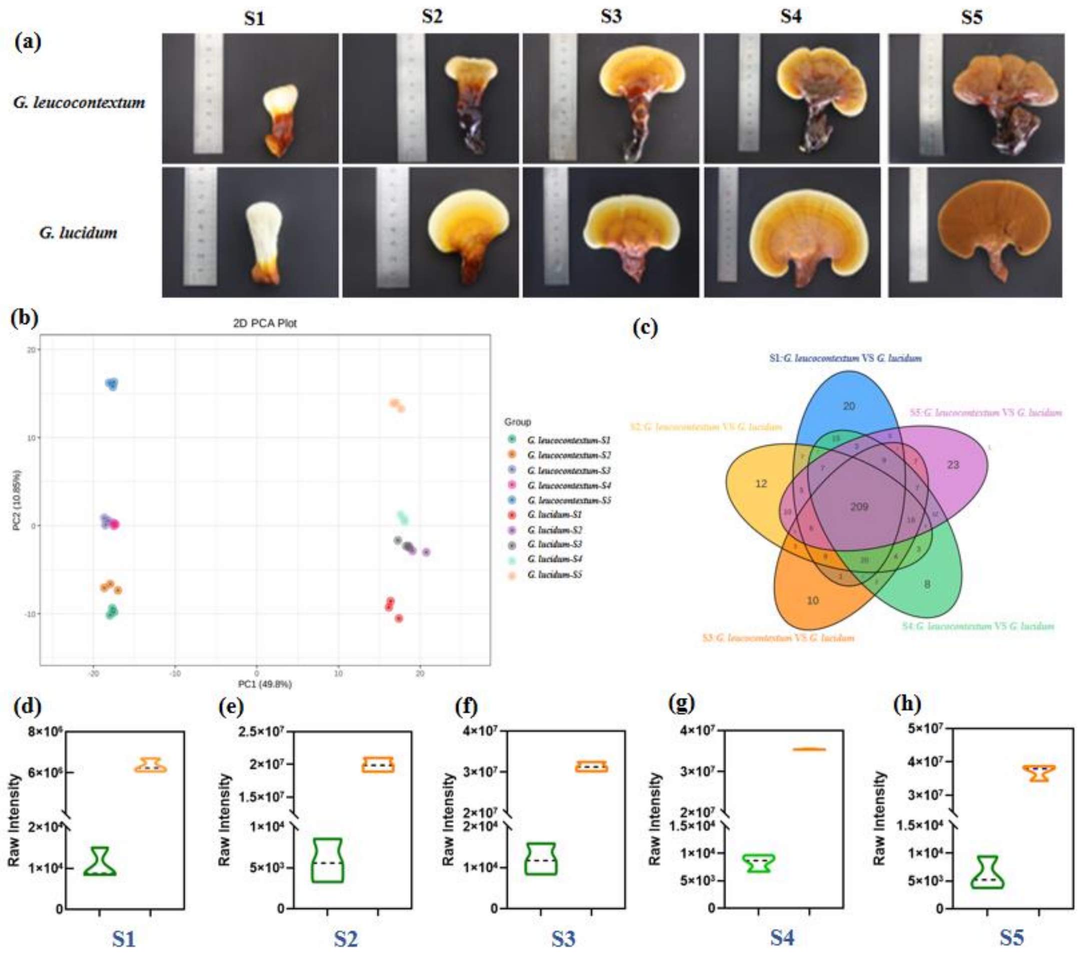

Fig1 (a) The phenotype of the sporocysts of *G. leucocontextum* and *G. lucidum* during five different developmental stages (S1: Budding stage; S2: Pileus differentiation stage; S3: Expansion stage; S4: Maturation stage; S5: Spore release stage). (b) Principal component analysis of metabolites during different developmental stages of *G. leucocontextum* and *G. lucidum*. (c) Venn diagram of metablite featuresdetected in sporocysts. (d~h) Violin box plots of ganoleuconin D content in G. leucocontextum and G. lucidum during the stages from S1 to S5. **Yellow**: *G. leucocontextum*; **Gre**en: *G. lucidum*

Table1 Differential metabolites of the major triterpenoids between *G. leucocontextum* and *G. lucidum*

Componds	S1		S2		S3		S4		S5	
	VIP	P value	VIP	P value	VIP	P value	VIP	P value	VIP	P value
Ganoderic acid G	1.17E+00	3.12E-05	1.16E+00	3.65E-04	1.15E+00	7.21E-04	1.15E+00	5.45E-04	1.14E+00	2.47E-03
Ganoderic acid K	1.17E+00	5.91E-04	1.16E+00	5.43E-03	1.15E+00	8.74E-04	1.15E+00	2.90E-03	1.14E+00	4.64E-03
Ganoderic acid F	1.16E+00	1.92E-03	1.16E+00	4.22E-04	1.15E+00	4.84E-03	1.15E+00	2.64E-04	1.14E+00	6.89E-04
Ganoderic acid C1	1.17E+00	1.03E-03	1.16E+00	9.83E-04	1.15E+00	1.25E-04	1.15E+00	2.35E-04	1.14E+00	3.79E-04
Ganodermic acid D	1.17E+00	2.28E-04	1.16E+00	4.90E-04	1.15E+00	2.76E-04	1.15E+00	4.68E-05	1.14E+00	1.60E-04
Ganodermic acid GS-2	1.17E+00	1.97E-04	1.16E+00	1.24E-04	1.15E+00	5.66E-04	1.15E+00	1.61E-03	1.14E+00	1.72E-04
Ganoleuconin L	1.17E+00	1.08E-03	1.16E+00	4.69E-04	1.15E+00	4.69E-04	1.14E+00	1.16E-03	1.14E+00	1.09E-04
Ganoleuconin J	1.16E+00	1.32E-04	1.16E+00	1.39E-03	1.15E+00	2.22E-03	1.15E+00	9.06E-04	1.14E+00	3.46E-04
Ganoleuconin D	1.16E+00	8.88E-04	1.16E+00	9.30E-04	1.15E+00	4.36E-04	1.15E+00	4.78E-06	1.14E+00	1.34E-03
Ganoleuconin K	1.16E+00	8.30E-04	1.16E+00	2.93E-05	1.15E+00	1.27E-03	1.15E+00	8.72E-04	1.14E+00	6.83E-04

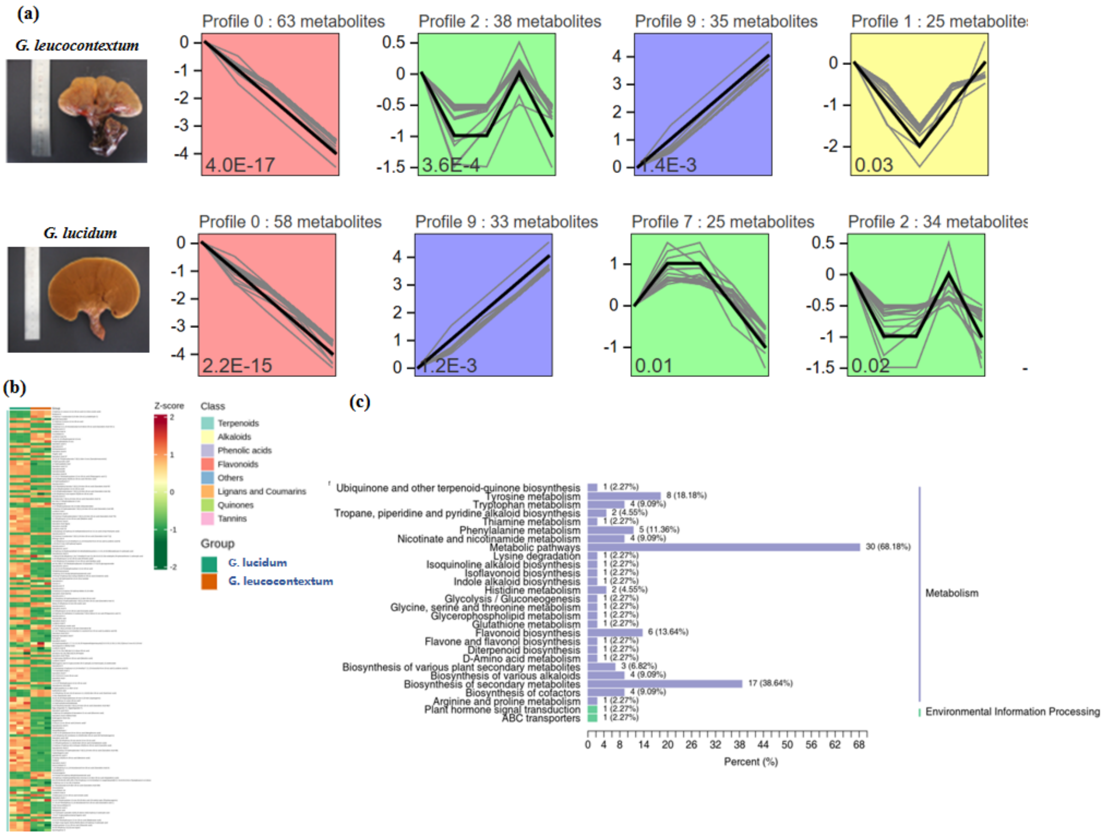

Fig2. (a) Trend analysis of all triterpene substances between *G. leucocontextum* and *G. lucidum*. (b) Secondary classification of the main differential metabolites of *G. leucocontextum* and *G. lucidum* during S5 stage. (c) KEGG analysis of major differential metabolites at S5 stage.

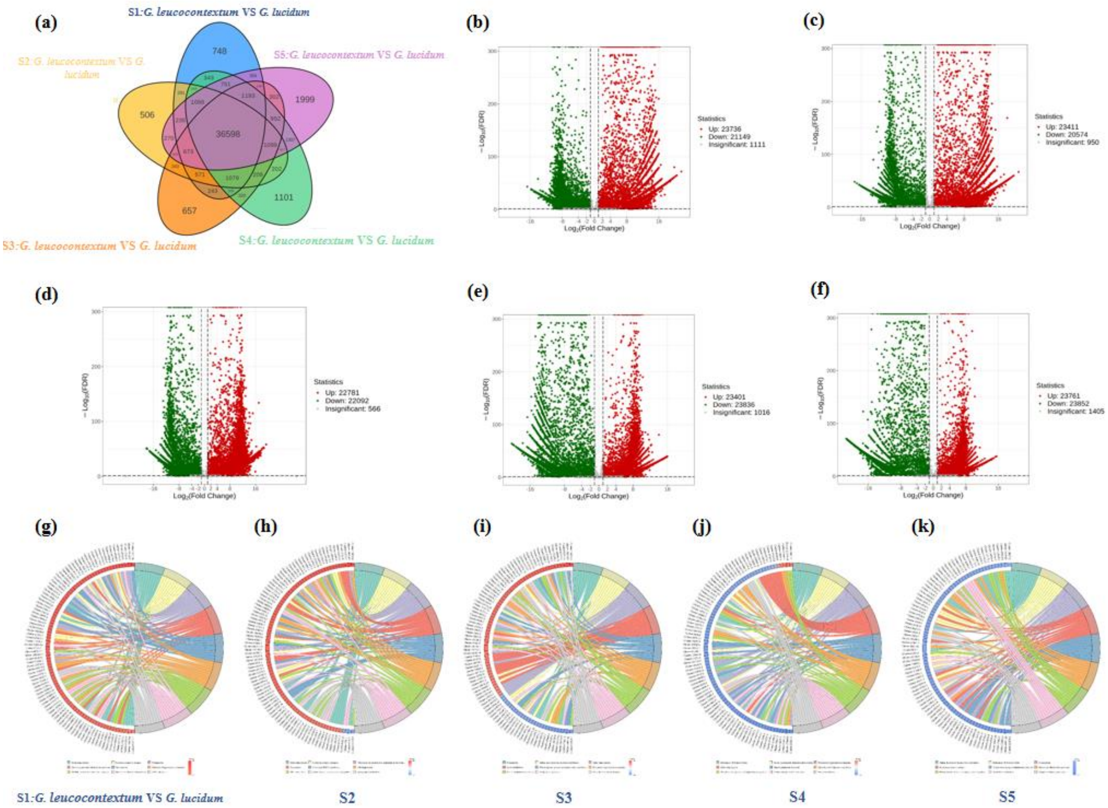

Fig3 The transcriptome analysis in different developmental stages. (a) The Venn diagrom of DEGs between *G. leucocontextum* and *G. lucidum* during the same developmental stage. (b~f) Volcano maps of up- and down-regulated genes in *G. leucocontextum* and *G. lucidum* from S1 to S5. (g~k) Statistics of KEGG pathway enrichment. The circos plot shows cluster IDs obtained by resequencing on the left, and pathways enriched for the top nine on the right

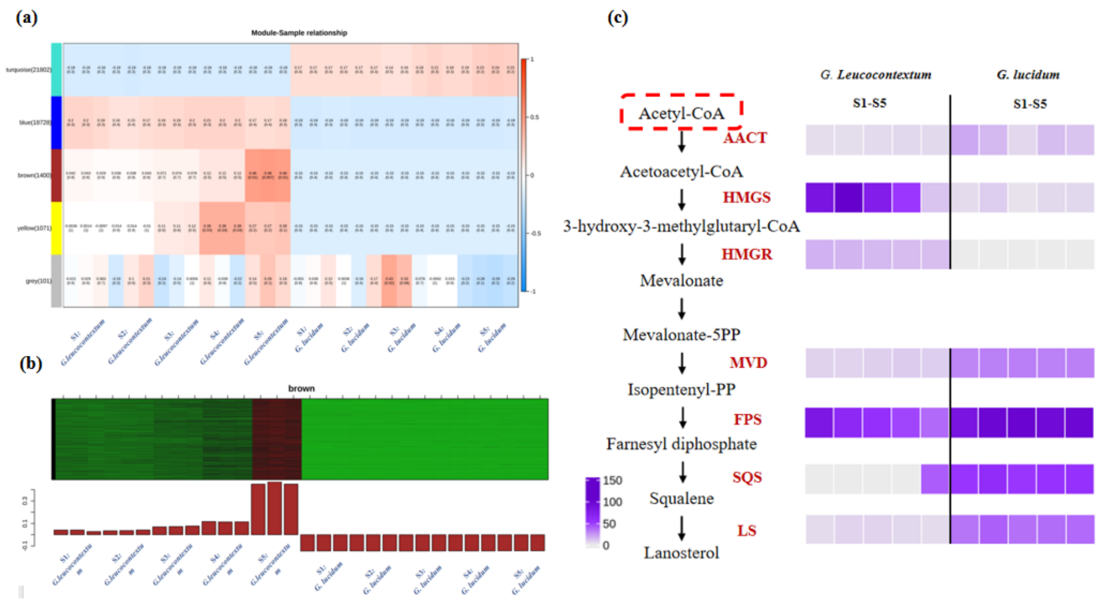

Fig4 (a) The weighted gene co-expression network analysis (WGCNA) of DEGs. Clustering dendrogram showing 21 modules of co-expressed genes on the basis of WGCNA. (b) Trends in the correlation of 1400 genes in the brown module with five developmental stages between G.leucocontextum and G. lucidum. (c) Heat map of the expression of genes related to ganoderic acid metabolic pathway from S1 to S5 stage.

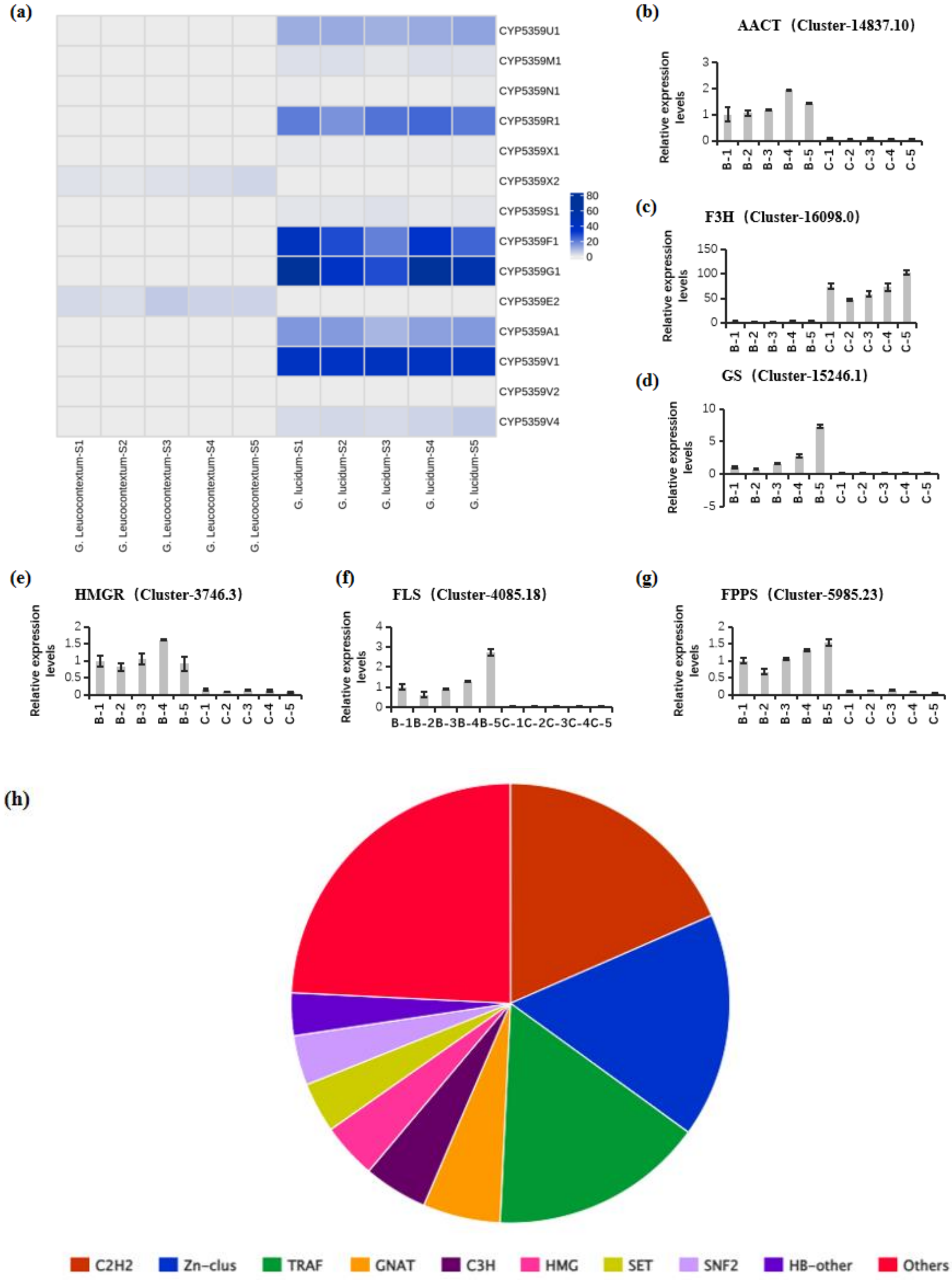

Fig5 (a) Heatmap of the expression of 14 arbitrarily selected genes specifically present in the CYP450s family of genes in Ganoderma. (b~g) The genes were screened for use in qRT-PCR experiments (which include the key upstream genes HMGR and AACT and the 2 downstream CYP450 family genes of the ganoderic acid metabolism pathway, 2 key flavonoid metabolism pathway genes FLS and F3H, and the glutamine synthetase gene GS). (h) Transcriptome sequencing screened a total of 2786 transcription factors.

References

Boh, B., Berovic, M., Zhang, J., et al, 2007. Ganoderma lucidum and its pharmaceutically active compounds[J]. Biotechnology Annual Review. 13(07), 265-301.

Chen, B., Tian, J., Zhang, J., et al, 2017. Triterpenes and meroterpenes from Ganoderma lucidum with inhibitory activity against HMGs reductase, aldose reductase and α-glucosidase[J]. Fitoterapia. 120, 6-16.

Chen, H., Zhang, J., Ren, J., et al, 2018. Triterpenes and meroterpenes with neuroprotective effects from Ganoderma leucocontextum[J]. Chem Biodivers. 15, e1700567. doi:10.1002/cbdv. 201700567.

Chen, S., Xu, J., Liu, C., et al, 2012. Genome sequence of the model medicinal mushroom[J]. *Ganoderma lucidum*. Nat Commun. 3, 913.

Chu, S., DeRisi, J., Eisen, M., et al, 1998. The Transcriptional Program of Sporulation in Budding Yeast[J]. Science. 282, 699-705.

Dean, Xia., Chunjuan, Liu., Shibo, Lv., et al, 2015. Research progress of plant histone acetyltransferases[J]. BIOTECHNOLOGY BULLETIN. 31(7), 18-25.

Gao, X., Qi, J., Ho, C. T., et al, 2020. Structural characterization and immunomodulatory activity of a water-soluble polysaccharide from Ganoderma leucocontextum fruiting bodies[J]. Carbohydr Polym. 249, 116874.

Gao, J. J., Min, B. S., Ahn, E. M., et al, 2002. New triterpene aldehydes, from Ganoderma lucidum and their cytotoxicity against murine and human tumor cells[J]. Chemical and Pharmaceutical Bulletin. 50, 837-840.

Henchel, K. L., Escalante-Semerena, J. C., 2015. Acylation of biomolecules in prokaryotes: a widespread strategy for the control of biological function and metabolic stress[J]. Microbiology and Molecular Biology Reviews. 79(3), 321-346.

Huang, Y. C., Niu, C. Y., Yang, C. R., et al, 2016. The heat stress factor *HSFA6b* connects ABA signaling and ABA-mediated heat responses[J]. Plant Physiology. 172(2), 1182-1199.

Hu, H., Liu, Y., Mo, W., et al, 2017. Isolation, characterization and anti-cancer activity of two Ganoderma leucocontextum strains (in Chinese)[J]. Acta Edulis Fungi. 24, 50-54.

Jacob, P., Hirt, H., Bendahmane, A., 2017. The heat-shock protein/chaperone network and multiple stress resistance[J]. Plant Biotechnol J. 15(4), 405-414.

Jing, Y., Lin, R., 2020. Transcriptional regulatory network of the light signaling pathways[J]. New Phytol. 227, 683-697.

Jonathan, S. G., Kigigha, L. T., Ohimain, E., 2008. Evaluation of the inhibitory potentials of eight higher Nigerian fungi against pathogenic microorganisms[J]. Afr. J. Biomed. Res. 11, 197-202.

Kassir, Y., Granot, D., Simchen, G., 1988. IME1, a positive regulator gene of meiosis in S. cerevisiae[J]. Cell. 52, 853-862.

Komoda, Y., Shimizu, M., Sonday, et al, 1989. Ganoderic acid and its dervatives as cholesterol synthesis inhibitors[J]. Chemical and Pharmaceutical Bulletin. 37(2), 531-533.

Li, T. H., Hu, H. P., Deng, W. Q., et al, 2015. Ganoderma leucocontextum, a new member of the G.lucidum complex from south western China[J]. Mycoscience. 56(1), 81-85.

Liu, H. T., Li, B., Shang, Z. L., et al, 2003. Calmodulin is involved in heat shock signal transduction in wheat[J]. Plant Physiol. 132(3), 1186-1195.

Liu, S-X, Liu C-L L. J-y, Zhang J-B, Shang L-e, Luo X-K, et al, 2020. Research progress on artificial cultivation and active components of Ganoderma leucocontextum (in chinese)[J]. Edible Fungi China. 39, 1-4, 16. doi:10.13629/j.cnki.53-1054.2020.04.001.

Liu, Y. N., Lu, X. X., Dai, C., et al, 2017. Phospholipase D and phosphatidic acid mediate heat stress induced secondary metabolism in Ganoderma lucidum[J]. Environmental Microbiology. 19(11), 4657-4669.

Li, C., Shi, L., Chen, D., et al, 2015. Functional analysis of the role of glutathione peroxidase (GPx) in the ROS signaling pathway, hyphal branching and the regulation of ganoderic acid biosynthesis in Ganoderma lucidum[J]. Fungal Genetics & Biology. 82, 168-180.

Liu, G., Wang, K., Kuang, S., et al, 2018. The natural compound GL22, isolated from Ganoderma mushrooms, suppresses tumor growth by altering lipid metabolism and triggering cell death[J]. Cell Death Dis. 9, 689.

Li, X., Xie, Y., Peng, J., et al, 2019. Ganoderiol F purified from Ganoderma leucocontextum retards cell cycle progression by inhibiting CDK4/CDK6[J]. Cell Cycle. 18, 3030-3043.

Liu, H. T., Sun, D. Y., Zhou, R. G., 2005. Ca2+and AtCaM3 are involved in the expression of heat shock protein gene in Arabidopsis[J]. Plant Cell Environ. 28(10), 1276-1284.

Liu, Y. N., Zhang, T. J., Lu, X. X., et al, 2017. Membrane fluidity is involved in the regulation of heat stress induced secondary metabolism in Ganoderma lucidum[J]. Environmental Microbiology. 19(4), 1653-1668.

Liu, R., Zhang, X., Ren, A., et al, 2018. Heat stress-induced reactive oxygen species participate in the regulation of HSP expression, hyphal branching and ganoderic acid biosynthesis in Ganoderma lucidum[J]. Microbiological Research. 209, 43-54.

Min, B. S., Nakamura, N., Miyashiro, H., et al, 1998. Triterpenes from the spores of Ganoderma lucidum and their inhibitory activity against HIV-1 protease[J]. Chemical and Pharmaceutical Bulletin. 46, 1607-1612.

Richter, C., Wittstein, K., Kirk, P. M., et al, 2015. An assessment of the taxonomy and chemotaxonomy of Ganoderma[J]. Fungal Divers. 71, 1-15.

Seo, G. S., Kirk, P. M., 2000. Ganodermataceae: nomenclature and classification. In: Flood, J., Bridge, P.D., Holderness, P. (Eds.), Ganoderma Disease of Perennial Crops[J]. CABI Publishing, Wallingford, UK. 3-22.

Seo, H. W., Hung, T. M., Na, M., et al, 2009. Steroids and triterpenes from the fruit bodies of Ganoderma lucidum and their anti-complement activity[J]. Arch. Pharm. Res. 32, 1573-1579.

Shiao, M. S., 2003. Natural products of the medicinal fungus Ganoderma lucidum: Occurrence, biological activities, and pharmacological functions[J]. The chemical Record. 3(3), 172-180.

Soccol, C. R., Bissoqui, L. Y., Rodrigues, C., et al, 2016. Pharmacological Properties of Biocompounds from Spores of the Lingzhi or Reishi Medicinal Mushroom Ganoderma lucidum (Agaricomycetes): A Review[J]. Int. J. Med. Mushrooms. 18, 757-767.

Pan, J., Liu, X., Shi, P., et al, 2021. Chemical Constituents and Antioxidant Activities in Vitro of Ganoderma leucocontextum[J]. Science and Technology of Food Industry. 42(9), 340-346.

Shi, L., Ren, A., Mu, D., et al, 2010. Current progress in the study on biosynthesis and regulation of ganoderic acids[J]. Applied Microbiology & Biotechnology. 88(6), 1243-1251.

Syed , K., Nelson, D. R., Riley, R., et al, 2013. Genomewide annotation and comparative

genomics of cytochrome P450 monooxygenases (P450s) in the polypore species Bjerkandera adusta, Ganoderma sp. and Phlebia brevispora[J]. Mycologia. 105(6), 1445-1455.

Syed, K., Shale, K., Pagadala, N. S., et al, 2014. Systematic identification and evolutionary analysis of catalytically versatile cytochrome P450 monooxygenase families enriched in model basidiomycete fungi[J]. PLoS One. 9, 86683.

Wang, K., Bao, L., Ma, K., et al, 2017. A novel class of alpha-glucosidase and HMG-CoA reductase inhibitors from Ganoderma leucocontextum and the anti-diabetic properties of ganomycin I in KK-A(y) mice[J]. Eur J Med Chem. 127, 1035-1046.

Wang, K., Bao, L., Xiong, W., et al, 2015. Lanostane triterpenes from the Tibetan medicinal mushroom Ganoderma leucocontextum and their inhibitory effects on HMG-CoA reductase and alpha-glucosidase[J]. J Nat Prod. 78, 1977-1989.

Wang, W. F., Xiao, H., Zhong, J. J., 2018. Biosynthesis of a ganoderic acid in Saccharomyces cerevisiae by expressing a cytochrome P450 gene from Ganoderma lucidum[J]. Biotechnol Bioeng. 115, 1842-1854.

Wang, Q., Xu, M., Zhao, L., et al, 2021. Transcriptome dynamics and metabolite analysis revealed the candidate genes and regulatory mechanism of ganoderic acid biosynthesis during liquid superficial-static culture of Ganoderma lucidum[J]. Microbial Biotechnology. 14(2), 600-613.

Xie, C., Yan, S., Zhang, Z., et al, 2020. Mapping the metabolic signatures of fermentation broth, mycelium, fruiting body and spores powder from Ganoderma lucidum by untargeted metabolomics[J]. LWT. 129, 109494.

Xiong, C., Chen, C., Chen, Z., et al, 2016. Potentiation of neuritogenic activity of Ganoderma leucocontextum on rat pheochromocytoma cells (in Chinese)[J]. Nat Prod Res Dev. 28, 1135-1138. 1143.

Xu, Y. N., Zhong, J. J., 2012. Impacts of calcium signal transduction on the fermentation production of antitumor ganoderic acids by medicinal mushroom Ganoderma lucidum[J]. Biotechnology Advances. 30(6), 1301-1308.

Yang, C., Li, W., Li, C., et al, 2018. Metabolism of ganoderic acids by a Ganoderma lucidum cytochrome P450 and the 3-keto sterol reductase ERG27 from yeast[J]. Phytochemistry. 155, 83-92.

Zhang, J. J., Ma, K., Han, J. J., et al, 2018. Eight new triterpenoids with inhibitory activity against HMG-CoA reductase from the medical mushroom Ganoderma leucocontextum collected in Tibetan plateau[J]. Fitoterapia. 130, 79-88.

Zhang, X., Ren, A., Li, M. J., et al. 2016. Heat Stress Modulates Mycelium Growth, Heat Shock Protein Expression, Ganoderic Acid Biosynthesis, and Hyphal Branching of Ganoderma lucidum via Cytosolic Ca2+[J]. Applied & Environmental Microbiology. 82(14), 4112.

Zhao, R. L., He, Y. M., 2018. Network pharmacology analysis of the anti-cancer pharmacological mechanisms of Ganoderma lucidum extract with experimental support using Hepa1-6-bearing C57 BL/6 mice[J]. J. Ethnopharmacol. 210, 287-295.

Zhao, J., Lawrence, C. D., Robert, V., 2005. Elicitor signal transduction leading to production of plant secondary metabolites[J]. Biotechnol. Adv. 23, 283-333.

1. The manuscript generally follows the standard IMRaD (Introduction, Methods, Results, Discussion) structure. This is the generally accepted and most effective structure for scientific manuscripts, which helps improve clarity, logical flow, and reader comprehension. The distinction between the Results and Discussion sections is blurred, with explanatory statements appearing in the Results section, which needs to be addressed. It is recommended to ensure a clear and consistent distinction between the objective presentation of experimental observations in the Results section and the explanatory analysis, hypothesis generation, and connections to existing literature in the Discussion section. This structural improvement will significantly improve the logical flow, scientific integrity, and overall readability of the manuscript, bringing it into line with standard journal publishing practices.

Thank you very much for your meticulous review and constructive suggestions on our manuscript. Regarding your concern about the blurred distinction between the Results and Discussion sections, we have taken it very seriously. We have carefully revised the manuscript to ensure a clear and consistent separation. In the Results section, we now solely focus on the objective presentation of our experimental observations, without any explanatory statements. Meanwhile, in the Discussion section, we have thoroughly conducted explanatory analyses, generated hypotheses, and made connections to the existing literature.

2. Recommended to be added a new table (Table 2. Summary of key differentially expressed genes (DEGs) in the triterpenoid biosynthesis pathway) in the manuscript, which can directly links the transcriptomic findings with the metabolomic results. By summarizing the expression levels of genes known to be involved in the triterpenoid biosynthesis pathway, it provides a molecular basis for the observed differences in metabolite accumulation.

We sincerely appreciate your professional advice. To better integrate the findings from transcriptomics with the metabolomics results, we have optimized the presentation of Figure 4c. This figure showcases a heatmap of the expression levels of key genes in the triterpenoid biosynthesis pathway during different developmental stages. Additionally, we have added corresponding descriptive content in both the Results and Discussion sections. We considered using a table but were concerned about potential overlap with Figure 4c.

Once again, thank you for your valuable suggestions.

3. Recommended to be added a new table (Table 3. Identified transcription factors that may be involved in triterpenoid metabolism) in the manuscript. The identification of transcription factors is an important new finding of this study. A dedicated table will clearly present these key regulatory elements in a concise and organized manner, making their identification prominent. Transcription factors are major regulators of gene expression and are the main targets for metabolic engineering or functional validation studies. Their clear listing provides a direct roadmap for future studies aimed at validating their precise role and manipulating their expression to enhance triterpenoid biosynthesis.

Thank you very much for your insightful comment regarding the identification of transcription factors—we fully agree that these factors, as a key novel finding of our study, deserve clear and prominent presentation to highlight their role in triterpenoid metabolism and provide actionable guidance for future research. Following your recommendation, we have added a new table to the manuscript. This table systematically organizes the six putative transcription factors we identified,

including their cluster IDs, respective families (HSF or GNAT), and brief notes on their potential association with triterpenoid biosynthesis. We believe this addition effectively addresses your suggestion by enhancing the clarity and impact of our findings on transcription factors. Once again, we sincerely appreciate your constructive feedback, which has helped strengthen the academic rigor and practical value of our manuscript.

4. There are many inaccurate and unsuccinct language expressions in the manuscript, which need to be carefully revised.
Thank you very much for pointing out the issue of inaccurate and unsuccinct language expressions in our manuscript. We have taken your advice seriously and conducted a thorough revision of the entire manuscript.

5. The figures and tables in this manuscript could be improved in terms of clarity, labeling, and overall presentation.
Thank you for your valuable comment on the clarity, labeling, and overall presentation of the manuscript’s figures and tables. We have carefully optimized these elements as suggested: we enhanced figure clarity by improving resolution and adjusting color contrast for better data distinguishability.

6. The manuscript contains inconsistencies in citation format (e.g., "Shiao MS 2003" vs. "Boh et al., 2007" vs. "Li X et al., 2019" vs. "PAN Jun et al., 2021" vs. "Syed, K., Nelson, D. R., Riley, R., et al, 2013").
Thank you for pointing out the inconsistencies in the citation format of the manuscript. We have thoroughly checked all literature citations throughout the text, unified the format (including standardizing the presentation of authors’ names, the use of punctuation such as commas, and the placement of publication years), and ensured that all citations adhere to a consistent style conforming to academic norms.

Dear Reviewer,

Thank you very much for your meticulous efforts in reviewing our manuscript—your detailed revisions have been incredibly valuable for enhancing the quality and academic rigor of our work.

We have carefully implemented all the changes you suggested. Specifically, we have supplemented the required references in the corresponding sections to strengthen the evidential basis of our arguments; polished the language of relevant passages to improve clarity, accuracy, and stylistic consistency; and addressed all other editorial adjustments you proposed. These revisions have been seamlessly integrated into the revised manuscript, ensuring full alignment with your recommendations.

Regarding the small number of questions you raised about the content of the paper. The primary objective of this study is to identify the main medicinal (bioactive) components and their associated key genes by comparing the transcriptomic and metabolomic data of *Ganoderma leucocontextum* (a newly reported species) from Tibet, China and Pakistan with those of the traditionally used medicinal *Ganoderma lucidum* at different developmental stages. This work aims to lay a foundation for subsequent in-depth research. Since this is a comprehensive comparative study, there are certain sections in the manuscript that cover relatively broad scopes (e.g., sections related to CYP450 enzymes and Ganoderma polysaccharides). To enhance the clarity of the manuscript and ensure our descriptions are easily understandable, we have added supplementary explanations in the corresponding sections to provide necessary context and elaboration.

Once again, we sincerely appreciate your time and effort in reviewing our manuscript. Your contributions have been invaluable in bringing our work to a higher standard.

Re: Spectrum01686-25R1 (**Integrative analysis of metabolome and transcriptome reveals new insights into formation and regulation of major components of *leucocontextum*and *G. lucidum***)

Dear Dr. Yuanzuo Lv:

Your manuscript has been accepted, and I am forwarding it to the ASM production staff for publication. Your paper will first be checked to make sure all elements meet the technical requirements. ASM staff will contact you if anything needs to be revised before copyediting and production can begin. Otherwise, you will be notified when your proofs are ready to be viewed.

Data Availability: ASM policy requires that data be available to the public upon online posting of the article, so please verify all links to sequence records, if present, and make sure that each number retrieves the full record of the data. If a new accession number is not linked or a link is broken, provide production staff with the correct URL for the record. If the accession numbers for new data are not publicly accessible before the expected online posting of the article, publication may be delayed; please contact ASM production staff immediately with the expected release date.

Publication Fees: For information on publication fees and which article types have charges, please visit ourwebsite. We have partnered with Copyright Clearance Center (CCC) to collect author charges. If fees apply to your paper, you will receive a message from no-reply@copyright.com with further instructions. For questions related to paying charges through RightsLink, please contact CCC at ASM_Support@copyright.com or toll free at +1-877-622-5543. CCC makes every attempt to respond to all emails within 24 hours.

ASM Membership: Corresponding authors may join or renew ASM membership to obtain discounts on publication fees. Need to upgrade your membership level? Please contact Customer Service at Service@asmusa.org.

Embargo Policy: A press release may be issued as soon as the manuscript is posted on theSpectrum Latest Articles webpage. The corresponding author will receive an email with the subject line "ASM Journals Author Services Notification" when the article is available online.

The ASM Journals program strives for constant improvement in our submission and publication process. Please tell us how we can improve your experience by taking this quick Author Survey.

Thank you for submitting your paper to Spectrum.

Sincerely,
Yuheng Yang
Editor
Microbiology Spectrum
